# EHF suppresses cancer progression by inhibiting ETS1-mediated ZEB expression

**DOI:** 10.1038/s41389-021-00313-2

**Published:** 2021-03-12

**Authors:** Kaname Sakamoto, Kaori Endo, Kei Sakamoto, Kou Kayamori, Shogo Ehata, Jiro Ichikawa, Takashi Ando, Ryosuke Nakamura, Yujiro Kimura, Kunio Yoshizawa, Keisuke Masuyama, Tomoyuki Kawataki, Kunio Miyake, Hiroki Ishii, Tomonori Kawasaki, Keiji Miyazawa, Masao Saitoh

**Affiliations:** 1 Department of Biochemistry, Graduate School of Medicine, University of Yamanashi, Yamanashi, Japan; 2 Department of Otolaryngology, Head and Neck Surgery, Graduate School of Medicine, University of Yamanashi, Yamanashi, Japan; 3 Center for Medical Education and Sciences, Graduate School of Medicine, University of Yamanashi, Yamanashi, Japan; 4 Department of Oral Pathology, Graduate School of Medical and Dental Sciences, Tokyo Medical and Dental University, Tokyo, Japan; 5 Department of Molecular Pathology, Graduate School of Medicine, The University of Tokyo, Tokyo, Japan; 6 Department of Orthopaedic Surgery, Graduate School of Medicine, University of Yamanashi, Yamanashi, Japan; 7 Department of Oral and Maxillofacial Surgery, Graduate School of Medicine, University of Yamanashi, Yamanashi, Japan; 8 Department of Neurosurgery, Graduate School of Medicine, University of Yamanashi, Yamanashi, Japan; 9 Department of Health Sciences, Graduate School of Medicine, University of Yamanashi, Yamanashi, Japan; 10 Department of Pathology, Saitama Medical University International Medical Center, Saitama, Japan

**Keywords:** Tumour heterogeneity, Cell migration

## Abstract

ETS homologous factor (EHF) belongs to the epithelium-specific subfamily of the E26 transformation-specific (ETS) transcription factor family. Currently, little is known about EHF’s function in cancer. We previously reported that ETS1 induces expression of the ZEB family proteins ZEB1/δEF1 and ZEB2/SIP1, which are key regulators of the epithelial–mesenchymal transition (EMT), by activating the *ZEB1* promoters. We have found that *EHF* gene produces two transcript variants, namely a long form variant that includes exon 1 (EHF-LF) and a short form variant that excludes exon 1 (EHF-SF). Only EHF-SF abrogates ETS1-mediated activation of the *ZEB1* promoter by promoting degradation of ETS1 proteins, thereby inhibiting the EMT phenotypes of cancer cells. Most importantly, we identified a novel point mutation within the conserved ETS domain of *EHF*, and found that *EHF* mutations abolish its original function while causing the EHF protein to act as a potential dominant negative, thereby enhancing metastasis in vivo. Therefore, we suggest that EHF acts as an anti-EMT factor by inhibiting the expression of ZEBs, and that *EHF* mutations exacerbate cancer progression.

## Introduction

Squamous cell carcinoma is the predominant tumor type in head and neck cancer. Approximately two-thirds of patients with head and neck squamous cell carcinoma (HNSCC) present local metastases in bones and regional lymph nodes during their first visit to a hospital, and are therefore diagnosed as advanced stages^1^. Within 2 years after various treatments, including surgery, chemotherapy, and radiotherapy, more than 50% of patients have local recurrence or distant metastasis, resulting in extremely poor prognosis and overall survival^1,2^.

The process of cancer cell invasion and metastasis requires the loss of cell–cell interactions combined with the acquisition of motility, occasionally undergoing epithelial–mesenchymal transition (EMT)^3–5^. These phenotypic changes are regulated by extracellular matrix components, exosomes, and soluble factors, which regulate several EMT transcription factors (EMT-TFs), including the ZEB family of two-handed zinc-finger factors (ZEB1 [Zinc-finger E-box binding homeobox 1]/δEF1 [δ-crystallin/E2-box factor 1] and ZEB2/SIP1 [Smad-interacting protein1]). Since EMT-TF gene mutations are rarely found in various cancers, EMT is thought to be a transient state, suggesting that cancer cells undergo a reversion process upon arriving at distant metastasized tissues called mesenchymal–epithelial transition (MET)^6,7^. ZEB1/2 (ZEB1 and ZEB2) protein levels, in particular, correlate positively with EMT phenotypes and aggressiveness of breast cancer cell lines^8^. We have previously reported that ETS1, one member of the E26 transformation-specific (ETS) family of transcription factors^9^, induces ZEB expression and activates the *ZEB1* promoter in breast cancer cells^10^. We have also reported that ETS1 silencing represses expression of ZEB1/2 and partially restores their epithelial phenotypes as well as their sensitivity to anti-tumor drugs.

The epithelium-specific ETS (ESE) transcription factors are a subgroup of ETS transcription factors defined by shared homology of the ETS domain, and include ELF3 (a.k.a. ESE1), ELF5 (a.k.a. ESE2), and EHF (a.k.a. ESE3)^11^. ELF3 is expressed in many different organs, whereas ELF5 and EHF expressions are restricted to glandular organs, including the salivary gland, mammary gland, and prostate gland. ESEs play crucial roles in normal development and have also been implicated in the pathogenesis of a wide range of cancers, playing both oncogenic and anti-oncogenic roles. In particular, *ELF3* gene amplification occurs in various kinds of cancers, and *ELF3* mutations frequently found in ampullary adenocarcinomas are heterozygous, suggesting that ELF3 may act as a haploinsufficient tumor suppressor^12^. However, the underlying mechanism of ELF3-mediated tumor suppression has not been elucidated. Recently, we found that ELF3 is highly expressed in the luminal subtype of breast cancer cells, and represses upregulation of ZEB1/2 by ETS1 in such cells^10^. Compared to *ELF3*, mutations in *EHF* are relatively rare in human cancers and are not frequently deposited in public datasets. Occasional mutation and amplification of *EHF* occurs in a subset of cancers, such as ovarian, stomach, and bladder cancer. However, little is known about the roles of EHF in cancer^11^.

In this study, we examined the roles of EHF in HNSCC cells. We found that EHF is expressed at low levels in mesenchymal-like HNSCC cells, whereas ZEB1/2 expression levels are extremely high compared to other epithelial-like HNSCC cells. The *EHF* gene encodes two transcript variants, a long form (EHF-LF) and a short form (EHF-SF) variant, which are produced independently by either inclusion or exclusion of the first exon. EHF-LF is deposited as the longest form or precursor form in the NCBI database. Surprisingly, EHF-SF is localized to nuclei, and could inhibit ETS1-induced activation of the *ZEB1* promoter by promoting degradation of ETS1 proteins, whereas EHF-LF is localized to the cytoplasm or perinuclei, and failed to inhibit ETS1-induced activation, due to difference in subcellular localization. Most importantly, an *EHF* point mutation identified in cancer cells caused a loss of the capacity to inhibit ETS1-induced phenomena, including upregulation of the ZEB1/2 and EMT phenotypes. We therefore propose that *EHF* mutation was first discovered to promote cancer aggressiveness by promoting EMT.

## Results

### EMT phenotypes in HNSCC cell lines

**Fig. 1:**
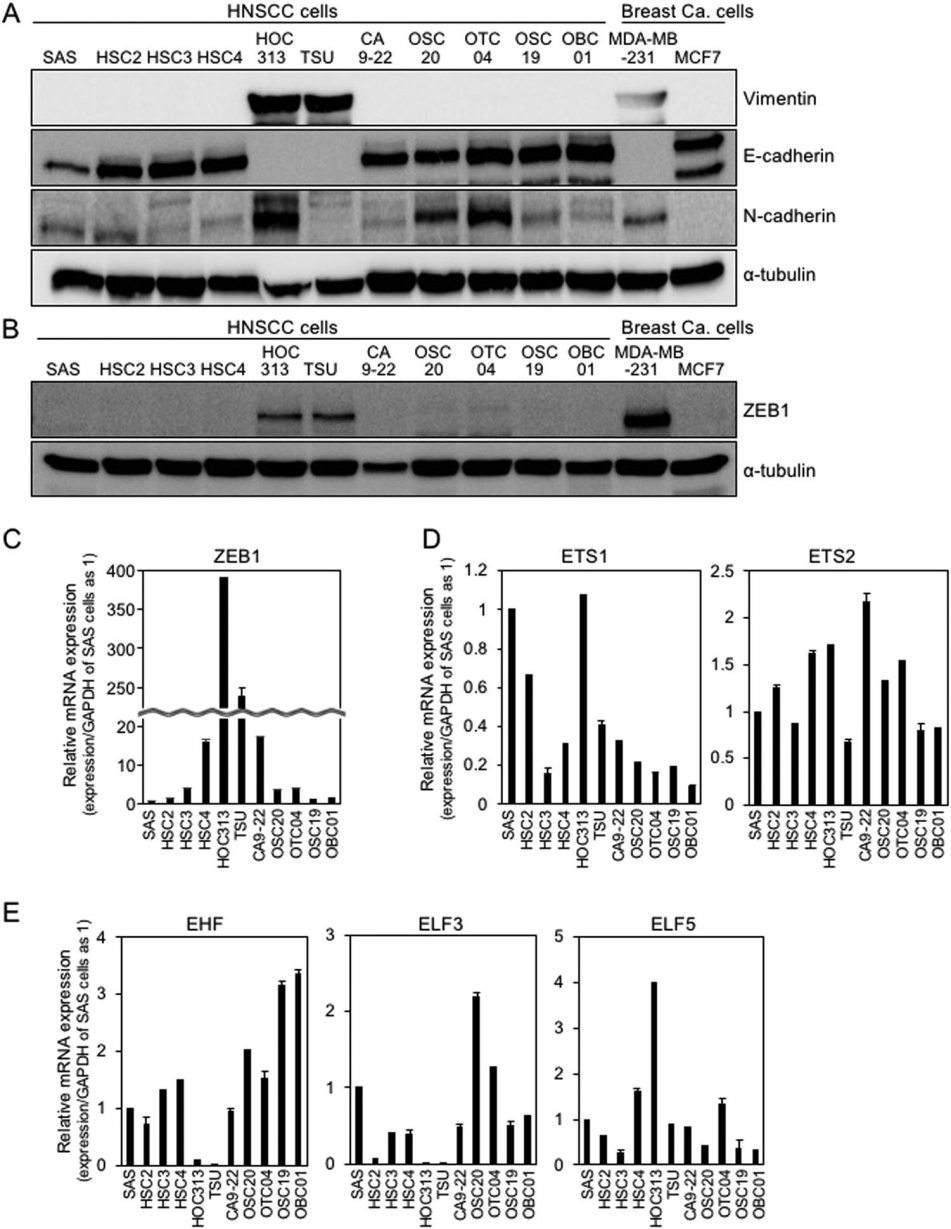
EMT phenotypes in HNSCC cells. **A, B, C, D, and E** Representative markers of EMT in HNSCC cells were determined by IB and RT-qPCR. Breast cancer MDA-MB-231 and MCF7 cells were used as controls for ZEB1 expression levels. α-tubulin was used as a loading control (**A** and **B**). The ratio of each mRNA to *GAPDH* in SAS cells was indicated as “1” (**C**, **D**, and **E**).

We previously reported that ZEB1/2 (ZEB1 and ZEB2) expression is positively correlated with the EMT phenotypes of breast cancer cell lines^8,13,14^. To determine the EMT phenotypes of HNSCC cells, we investigated 11 HNSCC cell lines by immunoblot (IB) analysis and included two subtypes of breast cancer cell lines as positive and negative controls. MDA-MB-231 cells are categorized into the basal-like subtype of breast cancer with high levels of ZEB1/2 expression and low levels of E-cadherin expression, whereas MCF7 cells are categorized into the luminal subtype with low levels of ZEB1/2 expression and high levels of E-cadherin expression^8,15^. Among the various HNSCC cell lines, we found that TSU and HOC313 cells, similar to MDA-MB-231 cells, express high levels of vimentin and ZEB1/2, and low levels of E-cadherin, while other HNSCC cells, similar to MCF7 cells, expressed high levels of E-cadherin and low levels of vimentin and ZEB1/2 (Fig. 1A, B and data not shown). Surprisingly, we found that N-cadherin is expressed nearly ubiquitously in HNSCC cells used in this study.

Using reverse transcription-quantitative PCR (RT-qPCR) analysis, we detected much higher levels of *ZEB1*/*2* mRNA in TSU and HOC313 cells than that in other epithelial-like HNSCC cells (Figs. 1C and S1A). Since we already reported that both ETS1 and ETS2 dramatically promote *ZEB1* promoter activation in breast cancer cells^10^, we determined mRNA levels of ETS family genes by RT-qPCR analysis in HNSCC cells. ETS1 and ETS2 mRNAs were detected in all HNSCC cells without positive correlation with ZEB1/2 expression (Figs. 1C, D, and S1A). However, *EHF* mRNA expression profiles showed the strongest inverse correlation with *ZEB1*/*2* in HNSCC cells (Fig. 1E). *ELF3* mRNA levels also exhibited an inverse correlation with *ZEB1*/*2*, though *ELF5* did not (Fig. 1E). These findings suggest that the EMT phenotypes of HNSCC cells are mediated by ZEB1/2, and that EHF regulates *ZEB1* expression without affecting mRNA levels of ETS in the mesenchymal-like HNSCC cells. In the Cancer Genome Atlas (TCGA) dataset of four oral squamous cell carcinoma tissues, *EHF* mRNA levels were not correlated negatively with those of ETS1 (Fig. S1B), while a weak negative correlation was detected only in the GSE37991 dataset (Fig. S1B). Notably, *EHF* mRNA levels in all four datasets were significantly lower in cancer tissues compared to normal tissues, which was correlated with overall survival (Fig. S1C, D).

### Two *EHF* transcript variants with different 5′UTRs

**Fig. 2:**
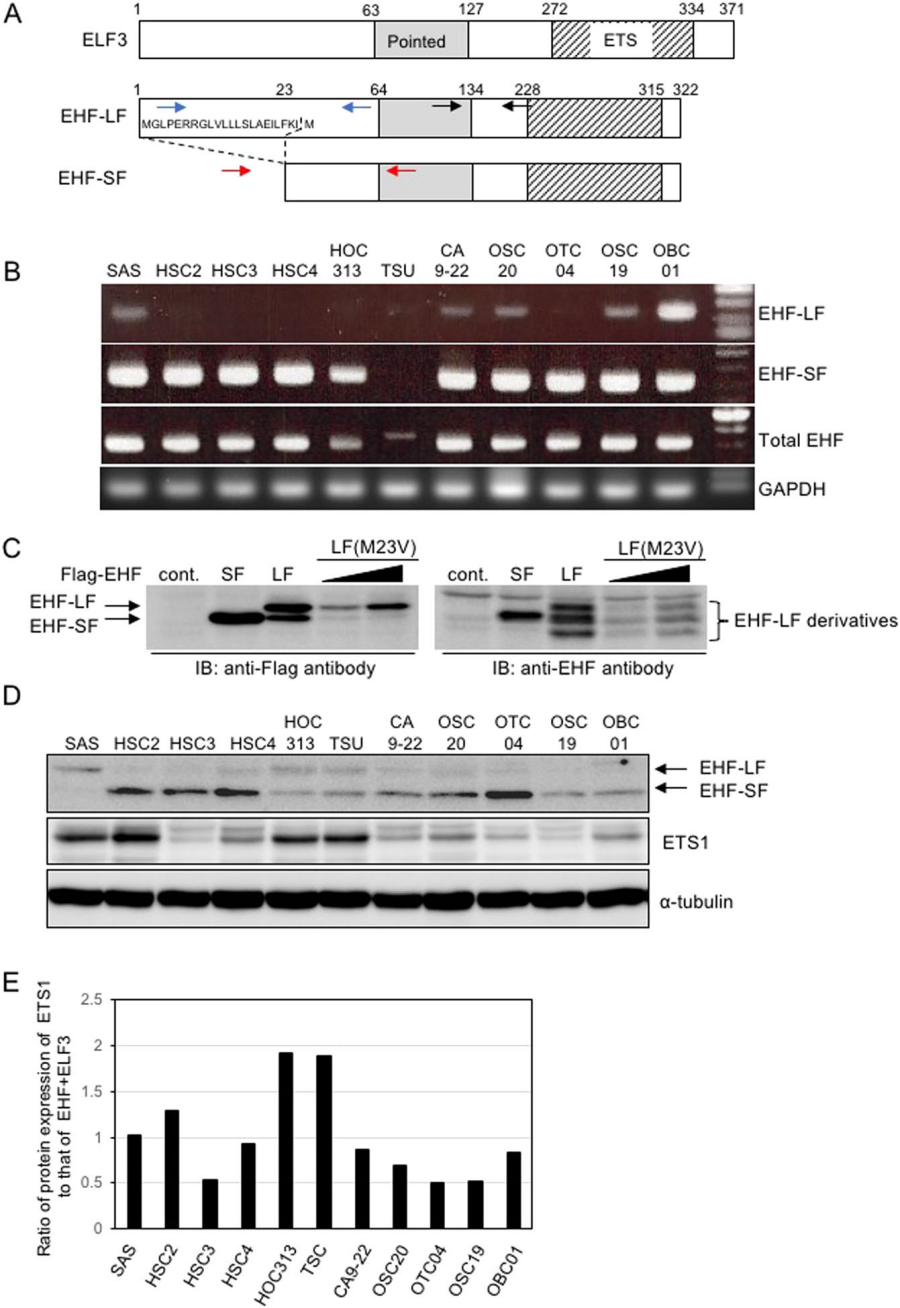
Two variants of EHF. **A** Schematic illustration of *ELF3* and *EHF* gene products is shown. The long form variant of EHF includes 22 amino acid residues from exon 1 (*EHF-LF*), whereas the short form variant of EHF excludes exon 1 (EHF-SF). The pointed and ETS domains are shown as grey and hatched areas, respectively. The primers used to amplify *EHF-LF*, *EHF-SF*, and total EHF are shown in blue, red, and black arrows, respectively. **B** mRNA levels of two *EHF* transcript variants were analyzed by conventional RT-PCR. **C** and **D** IB with the indicated antibodies were performed in COS7 transfected with control vector (cont.) or the indicated plasmids (**C**), and in HNSCC cells (**D**). **E** ETS1 (**D**), EHF-SF (**D**), and ELF3 (S2B) protein levels were densitometrically quantified and compared with those of α-tubulin. The ratio of protein expression of ETS1/α-tubulin to that of EHF-SF/α-tubulin + ELF3/α-tubulin in SAS cells was indicated as “1”. α-tubulin was used as a loading control (**D**). EHF-LF(M23V), a mutant in which Met 23 in EHF-LF was substituted with Val (**C**).

Although *EHF* is known to be similar in structure to *ELF3*, it produces two transcript variants: a long form variant that includes the first exon, EHF-LF, and a short form variant that does not, EHF-SF (Fig. 2A). First, we examined which variants were expressed in HNSCC cells by conventional RT-PCR analysis. *EHF-SF* mRNA was detected in almost all HNSCC cells, whereas *EHF-LF* mRNAs were detected in several cell types, including SAS, CA9-22, OSC20, OSC19, and OBC01 cells. Transcripts of both EHF variants were detected at low levels in TSU and HOC313 cells, respectively (Fig. 2B). Similar to the results of our RT-qPCR analysis (see Fig. 1E), total EHF levels were lower in mesenchymal-like HNSCC, TCU, and HOC313 cells (Fig. 2B). To determine whether the function of both variants is similar, we generated both *EHF-LF* and *EHF-SF* cDNAs using mRNAs isolated from human cancer cells. After transfecting COS7 cells with the plasmids that encode either flag-tagged EHF-LF or EHF-SF, we detected EHF-SF as a single band by SDS-PAGE and subsequent IB analysis using anti-flag and -EHF antibodies (Fig. 2C). By contrast, EHF-LF was detected with a few extra bands by both antibodies.

To exclude the possibility that flag-tagged EHF-LF generated a product that was translated starting from methionine 23 (Fig. 2A), Met 23 in EHF-LF was substituted with valine to generate EHF-LF (M23V). Similar to EHF-LF, EHF-LF(M23V) was detected as a few bands by IB analysis using anti-EHF antibody (Fig. 2C), suggesting that methionine residue at 23 was not available for translational start site to generate EHF-SF from the *EHF-LF* mRNA.

Because the anti-EHF antibody recognized both EHF protein variants, we could determine expression of endogenous EHF in various HNSCC cells. SAS, HOC313, and TSU cells exhibited slowly migrated band, which was not consistent with *EHF-LF* mRNA expression in HNSCC cells (Fig. 2B, D). Thus, we confirmed it by specific siRNAs against *EHF* (Fig. S2A), suggesting that the upper band in these cells was derived from *EHF-LF*. OTC04, HSC2, HSC3, and HSC4 cells expressed EHF-SF at high levels (Fig. 2D). Both mesenchymal-like HOC313 and TSU cells, which express high levels of ETS1 protein, expressed low levels of EHF and ELF3, (Figs. 1E, 2D and S2B), suggesting that the EMT-like phenotypes are highly dependent on the ratio of ETS1 protein levels to EHF and ELF3 protein levels (Fig. 2E).

### Only the EHF-SF variant suppresses *ZEB1* promoter activity

**Fig. 3:**
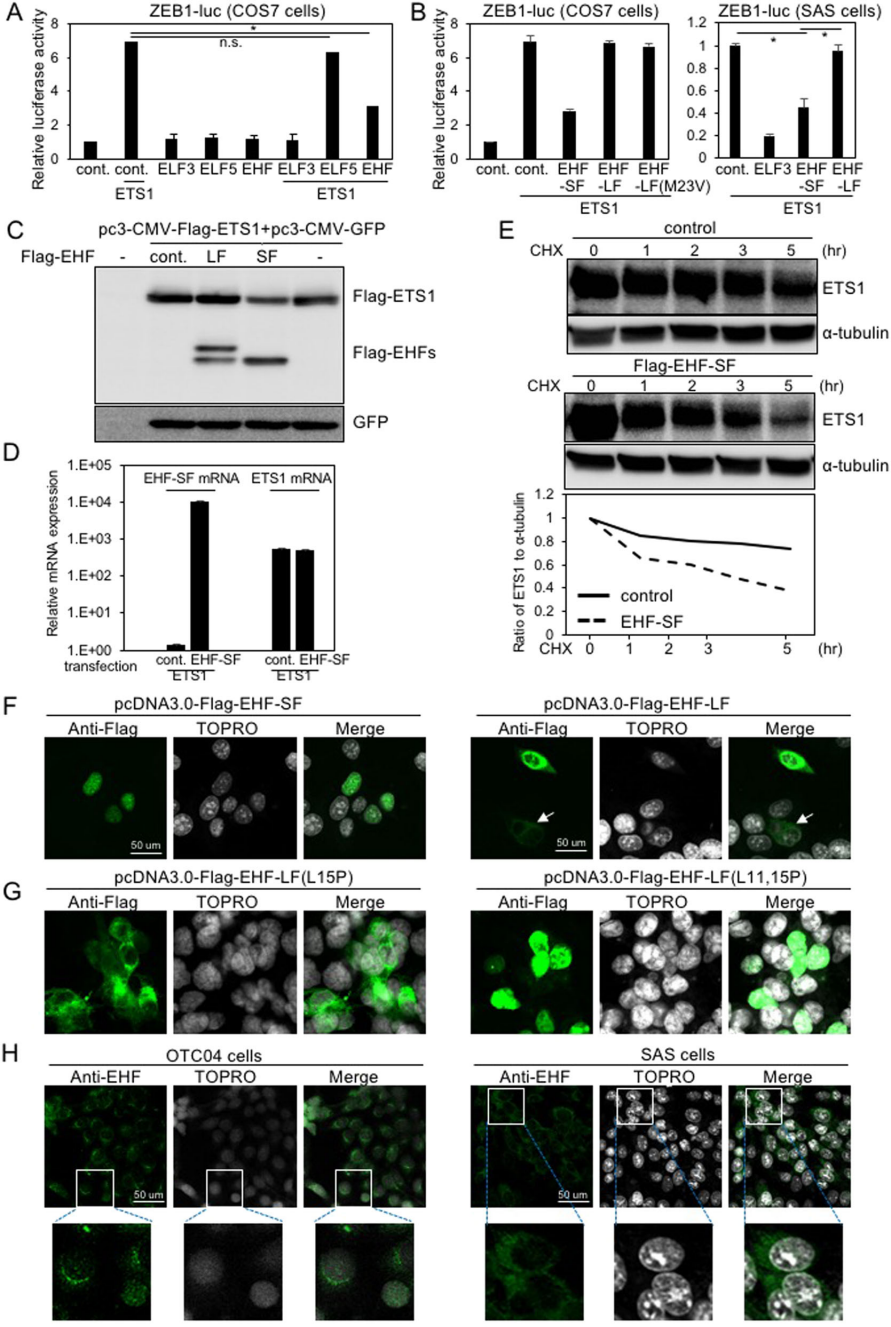
Inhibitory effects of EHF variants on ZEB1 promoter activity. **A** and **B** ZEB1 promoter activities were determined by luciferase assays after transfection with control vector (cont.) or the indicated plasmids. Luciferase activity of cells transfected with the control vector was indicated as “1.” Each value represents the mean ± s.d. of triplicate determinations from a representative experiment. Similar results were obtained in at least three independent experiments. *p* values were determined by Student’s *t*-test. *p < 0.01; n.s., not significant. (**C** and **D**) COS7 cells were transfected with the indicated plasmids, followed by IB (**C**) and RT-qPCR (**D**) to determine the levels of transfected genes. Ectopic *EHF-SF* mRNA levels of cells transfected with ETS1 alone was indicated as “1” (**D**). **E** COS7 cells pre-transfected with flag-tagged ETS1 were further transfected with either control vector or flag-EHF-SF plasmid, and treated with cycloheximide (CHX) for the indicated time, followed by IB. The ratio of ETS1 to α-tubulin was determined by densitometric analysis and shown at the bottom. (**F** and **G**) COS7 cells were transfected with the indicated plasmids followed by immunofluorescence analysis using an anti-flag antibody. Arrow indicates cells with low expression of flag-EHF-LF. **H** Subcellular localization of endogenous EHF was determined by anti-EHF antibody in HNSCC OTC04 and SAS cells. High magnification is shown in bottom panels.

To examine whether EHF affects *ZEB1* promoter activity, we transfected cells with plasmids carrying *EHF-SF*, *EHF-LF*, as well as *ELF3* in combination with *ETS1* plasmid, and determined *ZEB1* promoter reporter activity thereafter. As previously reported^10^, ETS1 dramatically activated the promoter’s activity, which was almost completely inhibited by ELF3 (Fig. 3A). EHF also partially inhibited the promoter activity induced by ETS1, whereas ELF5 failed to do so. Surprisingly, EHF-LF did not affect *ZEB1* promoter activity induced by ETS1, which was also observed in HNSCC SAS cells (Fig. 3B). EHF-LF (M23V) behaved similarly to EHF-LF, because Met 23 was not used as an initiation codon for translation to produce EHF-SF (Fig. 3B and see Fig. 2C). So far, it remains unclear how ELF3 inhibits *ZEB1* promoter activation by ETS1 in breast cancer cells^10^. To investigate the inhibitory effects of EHF-SF, we transfected EHFs together with both *GFP* and *ETS1*, both of which were subcloned into the same expression vectors under the control of the CMV promoter (pcDNA3.0). Overexpression of EHF-SF downregulated ETS1 protein levels in cells; however, overexpressed EHF-SF did not affect GFP protein levels. We also determined that EHF-SF downregulated ETS1 protein levels without affecting *ETS1* mRNA levels, whereas EHF-LF failed to affect either (Fig. 3C, D). In addition, we determined through experiments using cycloheximide that EHF-SF shortens the half-life of ETS1 proteins (Fig. 3E). We also found that siRNAs against *EHF* upregulated endogenous ETS1 proteins (Fig. S2C), which suggests that EHF-SF promotes the degradation of ETS1 proteins. Moreover, overexpressed ETS1 and ETS2 were downregulated in a manner dependent on the amount of EHF expression plasmids used for transfection (Fig. S2D).

Leucine-rich peptides are known to act as nuclear export signal/sequence (NES)^16^. We found that the first exon in *EHF-LF* is composed of 22 amino acid residues, including seven leucine and two isoleucine residues (see Fig. 2A). Upon transfection, EHF-SF and EHF-LF were clearly localized to the nucleus and cytoplasm, or to the perinuclear region, respectively, as determined by immunofluorescence using an anti-flag antibody (Fig. 3F). When leucine residues in the first exon of *EHF-LF* were substituted with proline residues, a double point mutant (L11,15P), but not a single point mutant (L15P), exhibited nuclear localization very similar to that of EHF-SF (Fig. 3F, G). This similarity suggests that leucine-rich residues in the first exon of EHF-LF regulate EHF subcellular localization. Also, the subcellular localization of EHF-LF in SAS cells differed slightly from that of EHF-SF in OTC04 cells (Fig. 3H). When the NES of EHF-LF was fused to EHF-SF (EHF-nesSF), the fusion protein was localized to the perinuclear cytoplasm and failed to degrade ETS1 proteins (Fig. S2E, F). Leptomycin B inhibits CRM1 (chromosomal region maintenance)/exportin 1, a protein required for nuclear export of proteins containing an NES^17^. Following treatment with leptomycin B, nuclear localized EHF-LF partially promoted the degradation of ETS1 proteins (Fig. S2E, G), suggesting that EHF nuclear localization involves degradation of ETS proteins.

### Biological significance of EHF in HNSCC cells

We next addressed the biological effects of EHF in HNSCC cells. At first, after TSU cells were infected with EHF-LF lentiviruses, we had difficulty detecting the EHF-LF protein despite the presence of the corresponding mRNA. The protein only became detectable following treatment with MG132, a proteasome inhibitor (Fig. S3A). In addition, endogenous EHF-LF proteins were upregulated in epithelial-like HNSCC cells following MG132 treatment (Fig. S3B). By contrast, when lentiviruses encoding EHF-SF were used for transduction, EHF-SF proteins were detected at moderately high levels even in the absence of MG132. MG132 treatment caused EHF-SF protein levels to increase slightly likely due to upregulation of its mRNA levels (Fig. S3C). Based on the finding that endogenous EHF-SF was easily detected by IB analysis in HNSCC cells (see Fig. 2D), endogenous EHF-SF protein is more stable in the nucleus than EHF-LF.

**Fig. 4:**
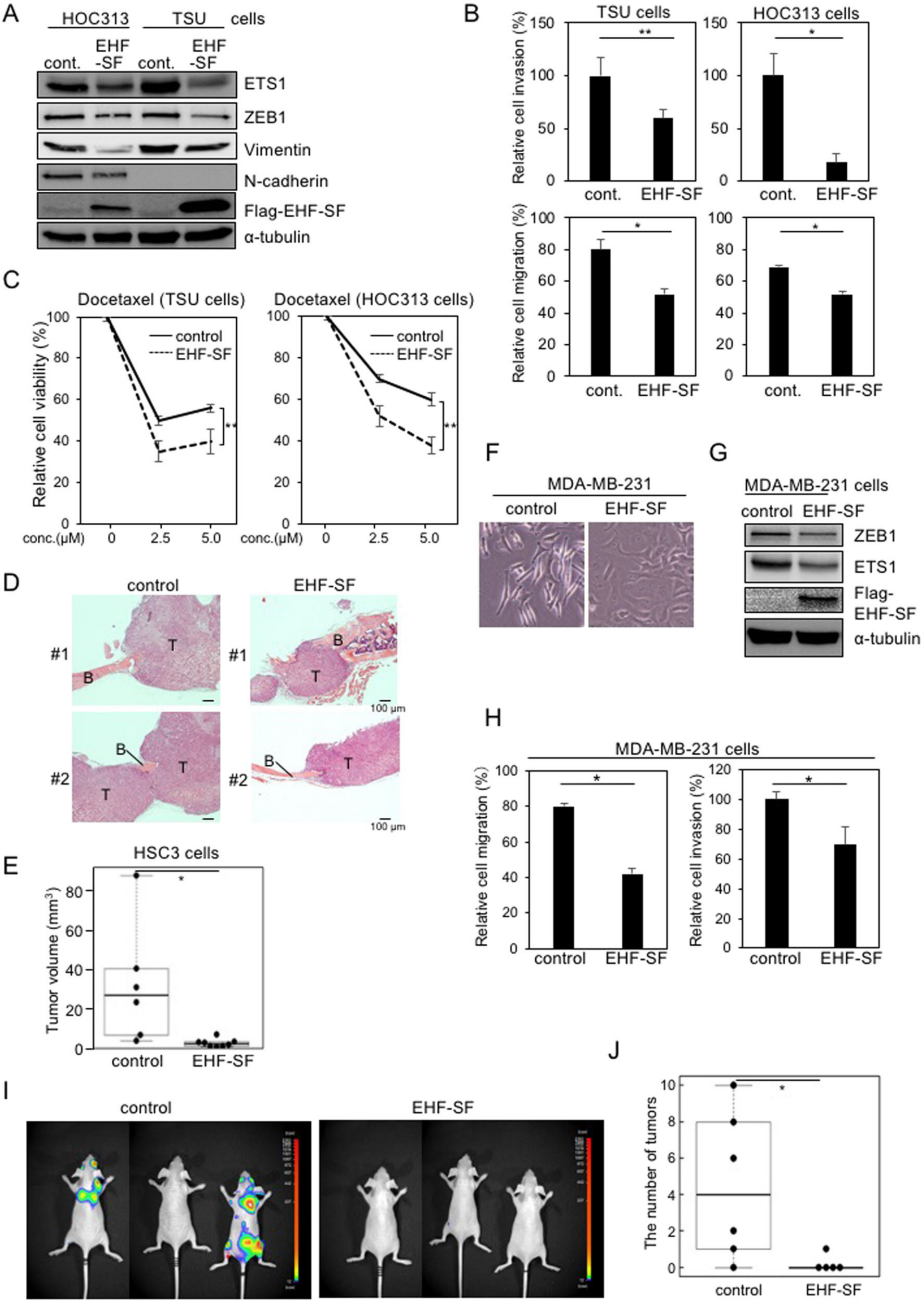
Inhibition of oncogenic effects of ETS1 by EHF-SF. **A, B**, and **C** HOC313 and TSU cells infected with lentiviruses carrying control (cont.) or flag-tagged EHF-SF were subjected to IB analysis using the indicated antibodies (**A**). Motility assays using a Boyden chamber assay with inserts coated with type I collagen gel (upper panels) and a wound healing assay (lower panels) (**B**). Chemoresistance assay in response to the indicated concentration (conc.) of docetaxel (**C**). Each value represents the mean ± s.d. of triplicate determinations from a representative experiment. Similar results were obtained in at least three independent experiments. **D and E** HSC3 cells infected with either control (*n* = 6) or flag-tagged EHF-SF (*n* = 8) were injected into the periosteal region of the parietal bone in mice. Five weeks later, mice were sacrificed, and tumor volume was measured (**E**). After decalcification, specimens were prepared. Typical histology in hematoxylin and eosin staining are shown. B bone, T tumor. (**F, G, H, I, and J**) MDA-MB-231-Luc cells infected with either control or EHF-SF were visualized by phase-contrast microscopy (**F**), and subjected to IB analysis (**G**) and motility assays (**H**). The cells were injected into the left ventricle of the heart, followed by imaging analysis (**I**) and quantification (**J**) at 35 days (5 weeks). *p* values were determined by Student’s *t*-test. *p < 0.01, **p < 0.03. α-tubulin was used as a loading control (**A** and **G**).

Following infection with *EHF-SF*, EHF-SF proteins were detectable in mesenchymal-like HOC313 and TSU cells in the absence of MG132 (Fig. 4A). In these lentivirus-infected cells, endogenous expression of both ETS1 and ZEB1 was repressed. In addition, vimentin was also downregulated by EHF-SF in both cell types, while N-cadherin was also repressed in HOC313 cells and barely detectable in TSU cells (Fig. 4A). Motility was suppressed by EHF-SF in both cell types according to migration and Boyden chamber assay, which was enhanced by sublethal concentration of docetaxel, an anti-cancer drug frequently used as a clinical therapeutic agent for patients with head and neck cancer (Figs. 4B, S3D, E)^18^. Sensitivity to docetaxel was significantly increased (Figs. 4C and S3F). Thus, these findings suggest that EHF inhibits EMT phenotypes, including motility and anti-cancer drug resistance, to some extent in HNSCC cells.

In vivo tumor growth was determined by a xenograft model involving transplantation of HSC3 cells into the calvarial region of mice^19^. Five weeks after transplantation, in vivo tumor growth was reduced by EHF-SF (Figs. 4D, E), while in vitro cell growth was not altered by EHF-SF overexpression in HSC3 cells (Fig. S3G). Since changes in the infiltration of the cells into calvaria (bone destruction) were not clearly evaluated by this experimental model, we went on to use breast cancer MDA-MB-231 cells to determine in vivo metastasis after intracardiac injection. We also chose this breast cancer cell line, because all the HNSCCs used in this study do not metastasize sufficiently to distant organs in mice. When EHF-SF was overexpressed in MDA-MB-231 cells, the cell morphology was altered from spindle-like to cobblestone-like shapes, a change that was accompanied by repression of *ZEB1* and *ETS1* as well as in vitro cell migration and anti-cancer drug resistance (Figs. 4F, G, H, and S3H). In vivo metastasis was also repressed by EHF-SF (Fig. 4I, J), suggesting that EHF-SF ameliorates the exacerbation of cancer in vivo.

### Tumor promotion in *EHF*-silenced cells

**Fig. 5:**
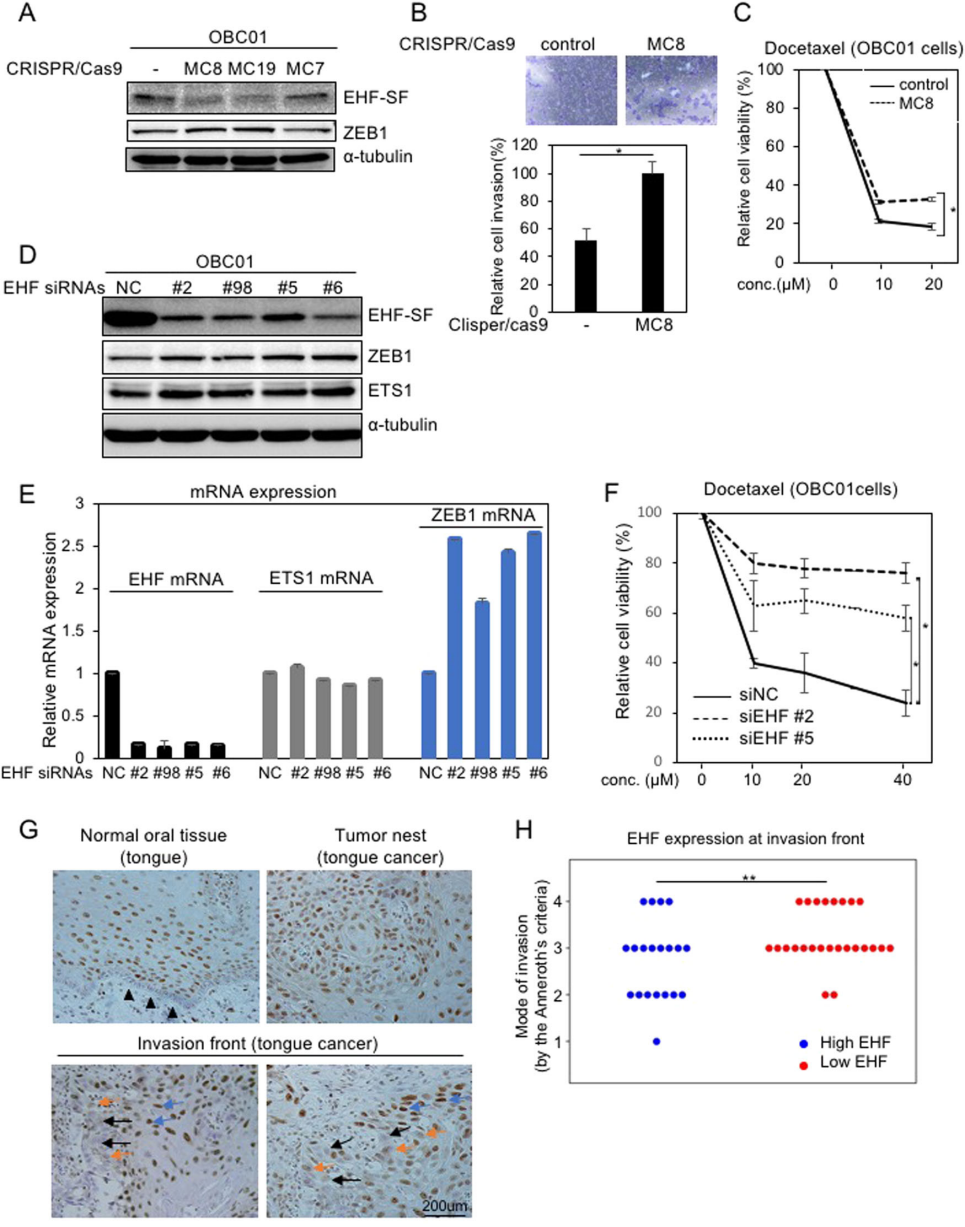
Effect of gene silencing of EHF in HNSCC cells. **A, B**, and **C** After knocking out the indicated genes by CRISPR/Cas9 techniques, monoclones were established and examined by IB analysis (**A**), invasion assays (**B**), and chemoresistance assays in response to the indicated concentration (conc.) of docetaxel in OBC01 cells (**C**). **D, E**, and **F** OBC01 cells transfected with control siRNA (NC) or EHF siRNAs were subjected to IB analysis (**D**), RT-qPCR analysis (**E**), and chemoresistance assays in response to the indicated concentration (conc.) of docetaxel (**F**). The ratio of mRNA expression to *GAPDH* in cells transfected with control siRNA (NC) was indicated as “1” (**E**). Each value represents the mean ± s.d. of triplicate determinations from a representative experiment. Similar results were obtained in at least three independent experiments. *p* values were determined by Student’s *t*-test. *p < 0.01 (**C** and **F**). α-tubulin was used as a loading control (**A** and **D**). **G** and **H** Representative images of immunohistochemical staining with anti-EHF antibody are shown in normal tongue tissue (top left panel), tumor nest (top right panel), and the invasion front (bottom two panels). Arrowheads indicate basal layer cells in normal tissue. Black arrows, orange arrows, and blue arrows represent EHF-negative, EHF-weak, and EHF-positive cancer cells at the invasion front, respectively (**G**). After representative images at the invasion front were randomly selected, we evaluated EHF-positive or negative/weak cancer cells at the invasion front, which were further assessed according to the Anneroth’s criteria by pathologists (**H**). Student’s *t*-test was used to compare differences between groups. **p < 0.02.

To perform loss of function experiments, we isolated several monoclones of HNSCC OBC01 cells after endogenous *EHF* was knocked out by CRISPR/Cas9 techniques using commercially available gRNA against the *EHF* gene. Although endogenous EHF-SF in these monoclones failed to be completely disrupted as determined by IB analysis, two monoclones exhibited moderate downregulation of EHF-SF and slight upregulation of ZEB1, leading to enhancement of motility and docetaxel resistance (Fig. 5A, B, C). In addition to the knockout cells, we generated OBC01 and HSC2 cells in which endogenous EHF expression was knocked down by specific siRNAs against *EHF*. The cells transfected with the siRNAs exhibited induction of *ZEB1* mRNA and ZEB1 protein expression. ETS1 protein levels were also increased without drastically affecting *ETS1* mRNA levels (Figs. 5D, E, and S4A). In addition, susceptibility to docetaxel was also reduced by *EHF*-specific siRNAs (Fig. 5F).

Immunohistochemical analysis was also performed using an anti-EHF antibody on specimens from human patients with tongue cancer after we characterized the specificity of the antibody using cells transfected with either siRNAs against EHF or plasmid encoding EHF-SF (data not shown). In the neighboring healthy tissues of patients with tongue cancer, EHF was predominantly stained in the nuclei in the cells of stratum spinosum, and hardly observed in basal layer cells (Fig. 5G, left in top panels). Some of the lymphoid cells also stained positive. In carcinoma tissues, EHF was observed in the nuclei of almost all cells in the tumor nest (Fig. 5G, right in top panels). Along with the invasion front, some cancer cells were either unstained or stained, coexisting with other cells that stained positive (Fig. 5G, bottom panels). Notably, statistical analysis showed that EHF was significantly reduced in cells at invasion front in high grade HNSCC (Fig. 5H). Therefore, EHF-SF acts as an anti-tumor promoting factor or anti-EMT factor in cancer.

### Identification of a novel point mutation in the *EHF* gene in cancer cells

**Fig. 6:**
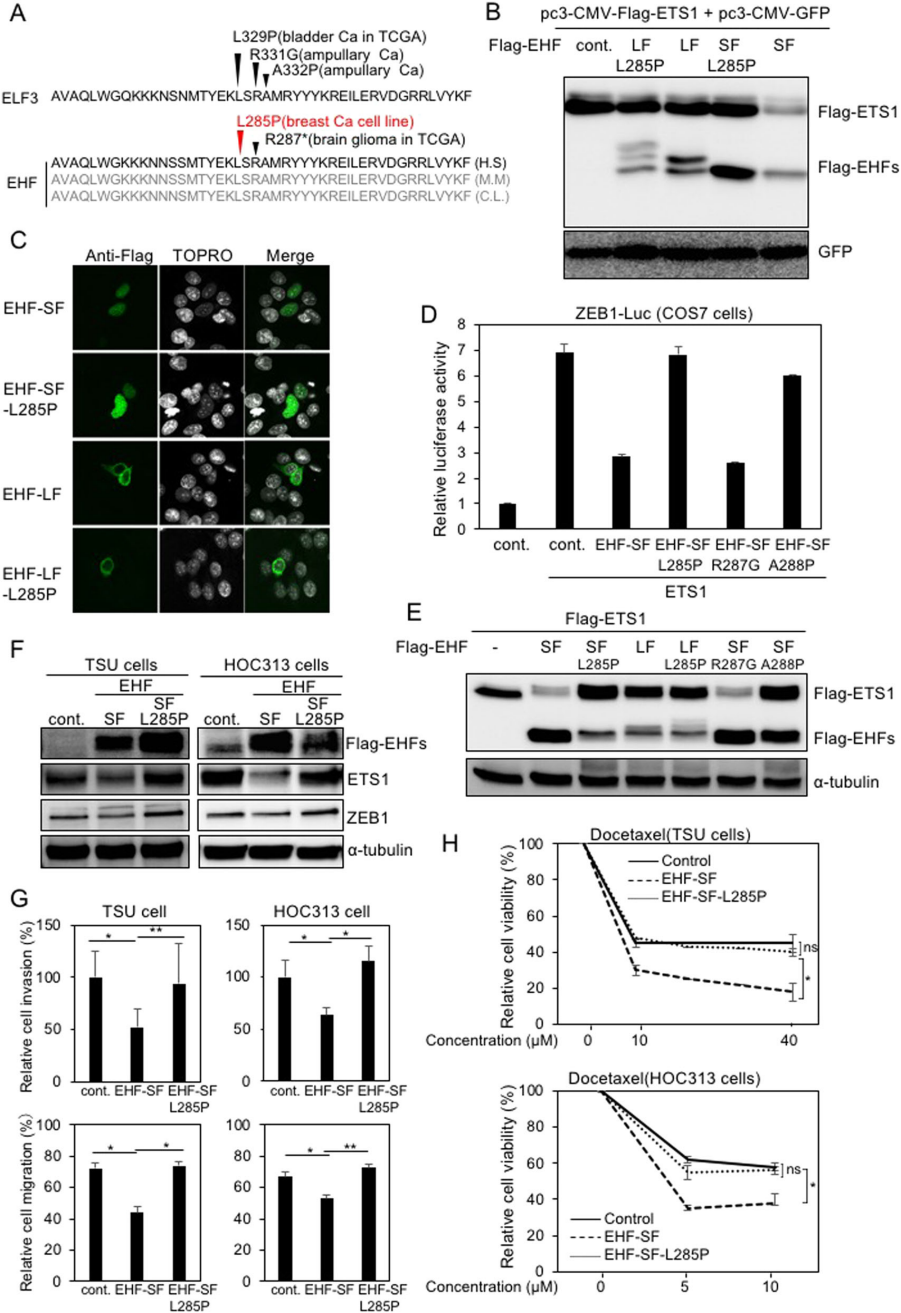
Identification of a point mutation in the *EHF* gene. **A** Amino acid comparison of a part of the ETS domain is shown in ELF3 (top) and EHF (bottom). Arrowheads indicate mutation previously annotated in the TCGA dataset or previous reports. Identification of a novel L285P mutation in EHF in breast cancer cells indicated with a red arrow. H.S *Homo sapiens*, M.M *Mus musculus*, C.L *Canis lupus*. **B, C, D**, and **E** Expression plasmids for EHF and its derivative mutants were transfected into COS7 cells and examined by IB (**B** and **E**), immunofluorescence (**C**), and luciferase analysis (**D**). **F, G**, and **H** TSU and HOC313 cells were infected with lentiviruses carrying control (cont.), flag-tagged EHF-SF, or flag-tagged EHF-SF-L285P, and subjected to IB analysis (**F**), motility assays using Boyden chamber assay with inserts coated with type I collagen gel (upper panels) and wound healing assays (lower panels) (**G**), and chemoresistance assays (**H**). Each value represents the mean ± s.d. of triplicate determinations from a representative experiment. Similar results were obtained in at least three independent experiments. *p* values were determined by Student’s *t*-test. ns not significant; *p < 0.01, **p < 0.03. α-tubulin was used as a loading control (**B**, **E**, and **F**).

When we generated *EHF* cDNAs using mRNAs isolated from breast cancer cells by RT-PCR in this study, we found that a leucine residue at 285 in EHF was mutated to proline (L285P) in several plasmid clones purified from bacteria. Proline 285 was restored to leucine by mutagenesis for use in the present experiments. Interestingly, we found that the region around position of 285 in ELF3, but not EHF, is a region where amino acid residues are frequently mutated in ampullary carcinoma (Fig. 6A)^12^. Since the L329P mutation in ELF3 is deposited in the TCGA dataset for bladder cancer (Fig. 6A), the corresponding L285P mutation in EHF was further investigated. Most surprisingly, the L285P mutation in EHF-SF abolished the effects of wild-type EHF-SF without affecting subcellular localization (Fig. 6B, C, D). We also constructed R287G and A288P mutants of EHF, which correspond to R331G and A332P, respectively, in the *ELF3* gene mutation in ampullary carcinoma (Fig. 6A)^12^. In addition, we constructed the R287* mutant of EHF, which is a nonsense mutation at position 287 deposited in the glioma TCGA dataset. The A288P and R287* mutations in EHF-SF failed to inhibit ETS1-induced activation of the *ZEB1* promoter and degrade ETS1, while the R287G mutant functioned similar to wild type EHF-SF (Figs. 6D, E, and S4B). When overexpressed from lentiviral vectors in TSU and HOC313 cells, which express endogenous EHF-SF only at low levels (see Fig. 2D), the EHF-SF-L285P mutation no longer downregulated *ETS1* and *ZEB1*, inhibited motility, and sensitized cells to anti-cancer drug (Fig. 6F, G, H). These finding strongly suggest that the L285 mutation results in loss of anti-EMT capacity of EHF-SF to inhibit EMT phenotypes.

### Tumor promoting effects of EHF-SF-L285P in vivo

**Fig. 7:**
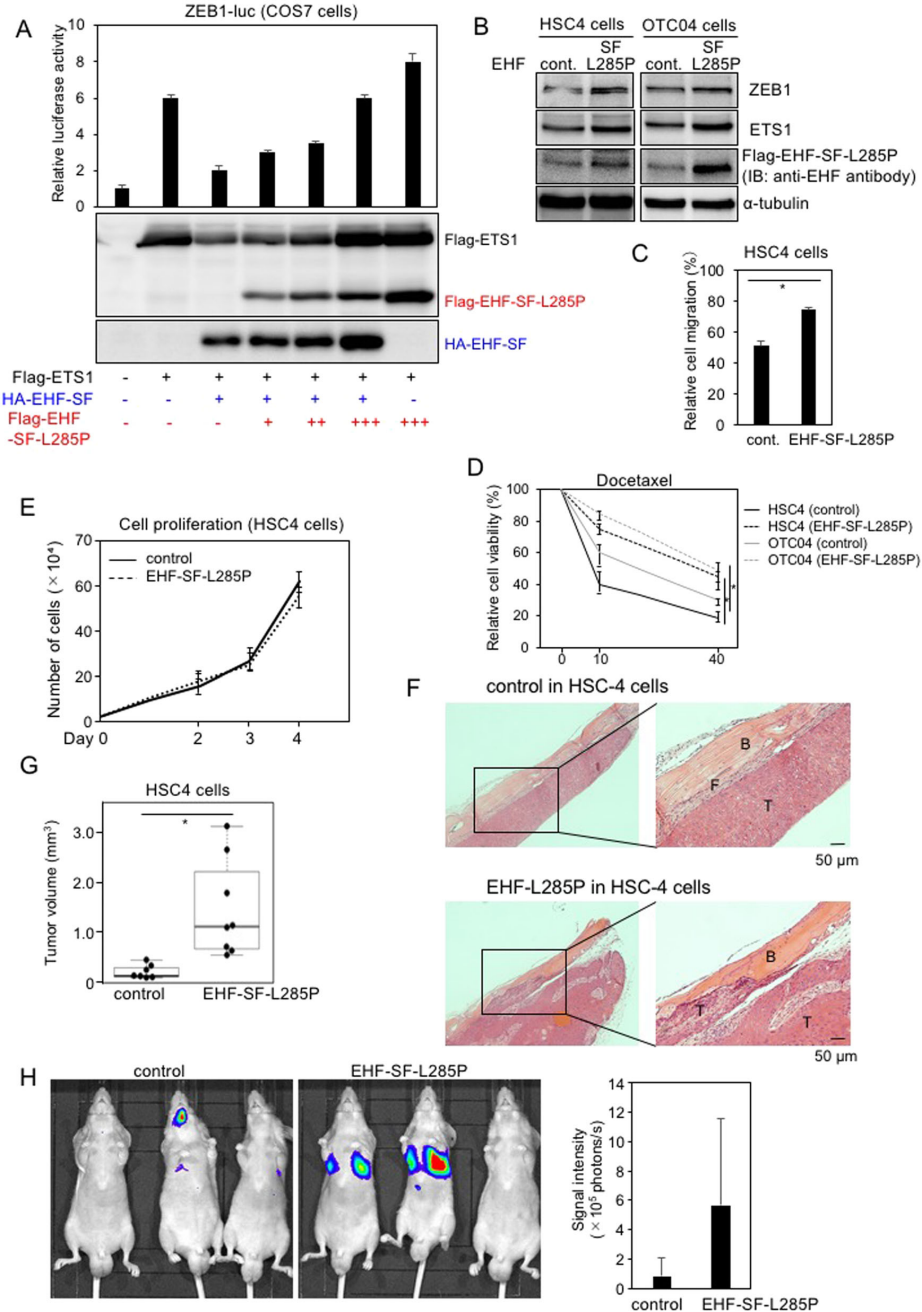
Effect of EHF-SF-L285. **A** *ZEB1* promoter activities were determined by luciferase assays (top panel), followed by IB (bottom panel), after cells had been transfected with the indicated plasmids. Each value represents the mean ± s.d. of triplicate determinations from a representative experiment. Similar results were obtained in at least three independent experiments. **B, C, D, E, F**, and **G** HSC4 and OTC04 cells were infected with either control (cont.) or flag-tagged EHF-SF-L285P and subjected to IB (**B**), motility assays (**C**), chemoresistance assays (**D**), and proliferation assays (**E**). HSC4 cells infected with lentiviruses carrying either control (*n* = 7) or flag-tagged EHF-SF-L285P (*n* = 8) were injected into the periosteal region of the parietal bone in mice. Five weeks later, mice were sacrificed and specimens were prepared after decalcification. Typical histology in hematoxylin and eosin staining are shown (**F**), followed by statistical analysis (**G**). T tumor, B bone, F fibrous tissue. *p* values were determined by Student’s *t*-test. *p < 0.01. **H** Human osteosarcoma 143B-Luc cells infected with either control (*n* = 5) or EHF-SF-L285P (*n* = 5) were injected intravenously into the lateral tail vein of mice. After mice were anesthetized with isoflurane on day 14, D-luciferin potassium salt was injected intravenously, followed by measurement of the emission intensity using the IVIS Lumina imaging system (left) and quantification analysis (right).

*ELF3* mutations are estimated to be a probability of heterozygous loss of function^12^. To determine if this is also a property of *EHF*, we first performed a titration analysis using expression plasmids encoding EHF-SF and EHF-SF-L285P in combination with the *ETS1* plasmid. Increased amounts of EHF-SF-L285P overcame the inhibitory effect of EHF-SF (Fig. 7A), which suggested that a dominant negative effect may be at work. Thus, we transduced epithelial-like HNSCC HSC4 and OTC04 cells, both of which express endogenous EHF-SF (see Fig. 2D), with EHF-SF-L285P lentiviruses. EHF-SF-L285P overexpression caused an increase in ETS1 and ZEB1 expression and promoted both migration and chemoresistance in both cell types (Fig. 7B, C, D). Cell proliferation in vitro was not dramatically affected by overexpression of EHF-SF-L285P (Fig. 7E). We next investigated the role of the L285P mutation in bone invasion into the calvarial region in a xenograft transplantation model. Unlike HSC3 cells (see Fig. 4), HSC4 cells are known to be less effective in bone invasion in this model than HSC3 cells (unpublished data). Five weeks after transplantation, HSC4 cells infected with EHF-SF-L285P exhibited enhanced tumor growth, compared to control cells. Histochemical analysis showed that control HSC4 cells adhered to the calvarial bone through periosteum-like mesenchymal tissues, while EHF-SF-L285P overexpression caused direct invasion into the bone and promoted tumor growth (Fig. 7F, G). These effects of EHF-SF-L285P were also determined using experimental pulmonary models with osteosarcoma 143B cells. After the cells were injected into the tail vein, the number of nodules in lung tissues increased with EHF-SF-L285P (Fig. 7H). These findings suggest that EHF-SF-L285P promotes tumor growth and invasion in vivo.

We further explored *EHF* mutations in human genomic DNA. Human genomic DNA was randomly extracted from 15 patients with tongue cancer by microdissection and analyzed by next generation sequencing of the ETS domain-encoding sequence in *EHF*. Unfortunately, we did not detect the L285P mutation, but we did detect mutations with missense, nonsense, and silent mutations in this region (Fig. S5). Notably, mutations in *ELF3* were not detected in these samples. Thus, EHF-SF, rather than ELF3, is associated with cancerization and progression of HNSCC.

## Discussion

In this study, we elucidated the functional differences between two EHF variants in cancer cells. Only EHF-SF, which is more widely expressed in HNSCC cells, can promote the degradation of ETS proteins, and, in turn, downregulate ZEB1/2, the most representative among EMT transcription factors (EMT-TFs). Therefore, we propose that EHF functions as an anti-EMT factor. Most importantly, the L285P mutation in EHF-SF abolishes its function and permits ETS1-mediated ZEB1/2 expression to promote EMT during cancer progression.

### Identification of the ESE domain in the anti-EMT function in cancer cells

The C-terminus of EHF (a.k.a. ESE3) contains an ETS domain (Fig. S5), though its amino acid sequence is only about 40% similar to that of ETS1. Hereafter, we call it the “ESE” domain, because ESE domain of EHF bears 85% amino acid similarity to that of ELF3 (a.k.a. ESE1) and 80% similarity to that of ELF5 (a.k.a. ESE2), respectively. A number of mutations in *ELF3* were identified in ampullary carcinoma by next generation sequencing analysis, many of which occur in the ESE domain, referred to as a hotspot region. Thus, *ELF3* is now known to be an anti-oncogene with potential haploinsufficiency^12^. Likewise, mutations in *ELF3* in many cancer tissues have also been deposited in the TCGA dataset, whereas those in *EHF* have not. In this study, we identified an EHF mutation that lies in the ESE domain (L285P) in breast cancer cells, which corresponds to the L329P mutation in ELF3 in bladder carcinoma (Fig. 6A). We also found mutations in genomic DNA within the ESE domain-encoding region of *EHF* in patients with tongue cancer (Fig. S5), which have not been deposited in TCGA or other databases. In addition, mutations around the L285 position in EHF were also deposited in the glioma TCGA dataset (Fig. 6A).

Notably, the molecular mechanism how *ELF3* mutants act as anti-oncogenes has not been elucidated so far^12^; however, we discovered that EHF-SF promotes degradation of the oncogene products ETS1 and ETS2, and subsequently reduces expression of ZEB1/2, acting as an anti-EMT factor. By contrast, the L285P mutation in EHF-SF impairs its intrinsic functions, suggesting that the ESE domain is indispensable for the anti-EMT effects of EHF-SF. Similar to EHF, ELF3 inhibited *ZEB1* promoter activation by ETS1 (Fig. 3A), and also promoted the degradation of ETS proteins. The L329P mutation, which corresponds to L285P in EHF, abolished its original function (Fig. S6A).

### Molecular mechanism of ETS1 degradation by EHF-SF

The precise molecular mechanism by which EHF-SF promotes ETS1 degradation remains unclear. ETS1 is known to be phosphorylated at specific serine and threonine residues by ERKs and CaMKII, some of which promotes ubiquitination by COP1 and sumoylation by UBC9 and PIASγ^20^. Phosphorylation of a neighboring tyrosine residue by Src family kinases can disrupt binding of COP1, stabilizing ETS1^21^. In our study, after immunoprecipitated ETS1 was blotted with anti-phosphotyrosine antibody (Py20), phosphorylated tyrosine was not clearly detected in either the presence or absence of EHF-SF or EHF-SF-L285P (data not shown). In addition, ETS1 degradation by EHF-SF was not blocked by several inhibitors against src (PP1), MEK1/2 (U0126), CaM kinase II (KN93), and PKC (Gö 6983) (Fig. S6F). A sumoylation inhibitor (2-D08) and lysosome inhibitor (chloroquine) also did not block ETS1 degradation (Fig. S5E and data not shown)^22^. In contrast, the proteasome inhibitors, MG132 and Lactacystin, inhibited ETS1 degradation induced by EHF-SF (Fig. S6B). In cells transfected with siRNAs against *EHF*, the stabilizing effects of MG132 on endogenous ETS1 protein were reduced (Fig. S6C), suggesting that endogenous EHF constitutively reduces ETS1 protein levels by proteasomal-dependent degradation. MG132 is a potent proteasome inhibitor with trileucine peptides (N-Benzyloxycarbonyl–L-leucyl–L-leucyl–L-leucinal), suggesting that the leucine repeat sequence (LVLLLSL) in the first exon of EHF-LF would behave like MG132. Thus, EHF-LF would act as a bait against unidentified molecules that degrade ETS proteins, resulting in stabilization of ETS proteins.

### Genomic alteration of the *EHF* gene

When we isolated *EHF* cDNAs from mRNAs of breast cancer cells, we accidentally found that a leucine residue at 285 in EHF was mutated to proline (L285P) in several clones. We therefore investigated genomic alteration in the ESE domain of the *EHF* and *ELF3* genes by sequencing, after genomic DNA was isolated from specimens of human patients with tongue cancer. Unfortunately, we could not detect the L285P mutation in *EHF* among 15 human patients with tongue cancer, though some mutations were found in the ESE domain (Fig. S5). Especially, an insertion was found at position 288 where a missense mutation (A332P) was detected in *ELF3* in ampullary adenocarcinomas. Surprisingly, genomic mutations of ELF3 were frequently found in ampullary adenocarcinomas and other cancers, while they were not detected in tongue cancer. The salivary gland is reported to have the high EHF expression in normal tissues^23^. Therefore, EHF, rather than ELF3, would be very involved in cancer progression in HNSCC.

## Materials and methods

### Cell lines

Eleven human HNSCC cells (SAS, HSC2, HSC3, HSC4, HOC313, TSU, CA9-22, OSC19, OSC20, OBC01, and OTC04), African green monkey kidney COS7 cells, human breast cancer MDA-MB-231 and MCF7 cells, and human embryonic kidney epithelial HEK293T cells were used in this study. HSC2, HSC3, and HSC4 were kind gifts from Dr. F. Momose and Dr. H. Ichijo^24,25^. CA9-22 was purchased from the Japanese Cancer Research Bioresources (Tokyo, Japan). Other HNSCC cell lines gifts from Dr. E. Yamamoto and Dr. S. Kawashiri as described previously^19^. HEK293T, HeLa, MDA-MB-231, MCF7, and COS7 were obtained from the American Type Culture Collection (Manassas, VA, USA). The cells were authenticated by Single Tandem Repeat analysis and cultured in DMEM (Nacalai tesque, Kyoto, Japan) supplemented with 10% heat-inactivated FBS, 500 units/ml penicillin, and 500 μg/ml streptomycin at 37 °C in a humidified atmosphere containing 5% CO_2_^26^. Cell culture supenatants are tested for mycoplasma contamination using TaKaRa PCR Mycoplasma Detection Set (Takara-Bio, Kusatsu, Japan).

### DNA constructs

The human *ZEB1* promoter reporter (hZEB1-Luc) and expression plasmids encoding human ELF3 were previously described^10,14^. cDNAs of *ELF5* and *EHF-LF* were synthesized by a PCR-based strategy using cDNA prepared from breast cancer cells. *EHF-SF* was constructed by a PCR-based strategy using *EHF-LF* as a template. Other plasmids used are described elsewhere^10^. Point mutations in EHF were introduced by PCR-based mutagenesis. All constructs were subcloned into the pcDNA3.0 expression vector with a flag tag at the N-terminus and confirmed by sequencing. The primers used for cloning are listed in Table S1.

Additional information on reagents and experimental procedures are described in the Supplementary Materials and Methods.

## Supplementary information

Supplemental information
