# Supplementary material for: EHF suppresses cancer progression by inhibiting ETS1-mediated ZEB expression
Source: Oncogenesis. 2021 Mar 12;10(3):26. doi: 10.1038/s41389-021-00313-2 (PMC7955083; doi:10.1038/s41389-021-00313-2)
Supplement: Supplementary file 1 — Supplemental information [file 41389_2021_313_MOESM1_ESM.pdf]

Supplementary Information

Supplementary Materials

Reagents and antibodies

MG132 and U0126 were obtained from CALBIOCHEM® (Darmstadt, Germany). Lactacystin was purchased from EMD Millipore Corp. (Billerica, MA.). 2-D08, PP1, KN93, chloroquine, and leptomycin B were purchased from Sigma-Aldrich (St. Louis, MO.). Docetaxel was purchased from Pepro Tech (Rocky Hill, NJ, USA). Rabbit polyclonal anti–ZEB1 and –ETS1 antibodies were from Novus Biologicals (Littleton, CO, USA) and Santa Cruz Biotechnology (Dallas, TX.), respectively. Rabbit polyclonal anti–EHF antibody was from Invitrogen (Carlsbad, CA). Rabbit monoclonal anti–ELF3 antibody was from Abcam (Cambridge, UK). Mouse monoclonal anti–E-cadherin and anti–N-cadherin antibodies were from BD transduction laboratories (Lexington, KY.). Rat monoclonal anti–HA, and mouse monoclonal anti–α-tubulin and anti–Flag antibodies were from Sigma-Aldrich. Rabbit monoclonal anti–vimentin antibody was from Cell Signaling Technology (Beverly, MA.).

Immunoblotting, immunofluorescence labeling, luciferase assay, and lentiviral infection

The procedures used for immunoblotting, immunofluorescence, and luciferase activity assays were previously described [1]. For production of lentiviral vectors, HEK293FT cells were transfected using Lipofectamine2000 with pCAG-HIVgp and pCMV-VSV-G-RSV-Rev vectors [2]. The culture media were collected 72–96 h after transfection and used for infection into cells.

RNA extraction and reverse transcription

Total RNA was extracted using the RNeasy Mini Kit with DNase treatment (Qiagen, Venlo, Netherlands), and stored at -80 °C until use. Purity of the RNA samples was assessed spectrophotometrically by measuring the OD_{260}/OD_{280} ratio. Reverse transcription was performed immediately following quality control assessment. Two micrograms of total RNA were reverse-transcribed into cDNA using the PrimeScript First Strand cDNA synthesis kit (Takara-Bio, Kusatsu, Japan).

Conventional PCR

Target sequence for conventional PCR primers was 300–500 bp long with a melting temperature of 55–65 °C and a GC content between 45% and 55%. PCR primers were designed using Primer-Blast. Conventional PCR was performed with LA Taq polymerase (TaKaRa). All PCR performed included an initial denaturation for 2 min at 95 °C. Amplification was performed for 25 cycles using the following parameters: 95 °C for 1 min, 98 °C for 20 s, 50 °C for 1 min, and 72 °C for 2 min, followed by an extension of 10 min at 72 °C. PCR products were separated on 2% agarose gels, stained with ethidium bromide, and visualized using a Printgraph AE-

6932GXES gel detection system (ATTO Corp. Tokyo, Japan). The *GAPDH* gene was used as an internal control in conventional PCR.

Quantitative real-time PCR (qPCR)

The target sequence for quantitative real-time PCR (qPCR) was 75–150 bp long with a melting temperature of 55–65 °C and GC content between 45% and 55%. PCR primers were designed using Primer-Blast. qPCR was performed using Power SYBR Green PCR Master Mix (Applied Biosystems, Foster City, CA) using a Step One Plus thermocycler with fluorescence detection (Applied Biosystems). The relative expression level of each mRNA was normalized against the level of *GAPDH* mRNA [3]. Gene expression levels were measured by relative qPCR. A standard curve was always used and was first generated by producing a two-fold dilution series over five points of the most concentrated cDNA sample [4, 5]. For each dilution, qPCR was performed in triplicate for all PCR primer pairs. The standard curve was constructed by plotting the log of the starting quality of template against the Threshold Cycle (Ct) values obtained. The equation of the linear regression line was used to determine whether the qPCR assay was optimized. qPCR detection instruments including 96-well plates were obtained from Applied Biosystems. The PCR primers are listed in Table S1.

To perform reverse transcription-qPCR (RT-qPCR), cells were seeded in two wells of the tissue culture plate. mRNA was extracted independently from cells, and each sample was split into three wells of a 96-well plate in order to measure endogenous mRNA levels by RT-qPCR. We performed these experiments at least twice, and representative results are shown

Small interfering RNA

Transfection of cells with siRNAs was performed using Lipofectamine RNAiMAX (Invitrogen). The siRNAs used in this study were: human ESE3 [HSS120049 (#2, Invitrogen), 4392420 (#98, Thermo Fisher Scientific, Waltham, MA), 05-0002 (#5, Dharmacon, Lafayette, CO), 06-0002 (#6, Dharmacon), and control (1157019, Invitrogen).

CRISPR/Cas9 experiments

Target genes in cells were knocked out out using Lipofectamine™ CRISPRMAX™Cas9 and TrueCut™ Cas9 Protein v2 (Invitrogen). EHF gRNA was used with TrueGuide Modified Synthetic sgRNA [CRISPR821048_SGM] (Invitrogen). Cells transfected with gRNA were mono-cloned and used for further experiments.

Wound healing assay

Cells were seeded in 6-well plates. After cells reached confluency, cells were scratched with a 10 µl disposable

pipet tip. Migration of wound edges was measured at five points on photographs acquired using an ECLIPSETS100 (Olympus, Tokyo, Japan), and the cell migration distance after 12 h was compared with the distance at 0 h.

Cell invasion and proliferation assay

Boyden chamber migration assays were conducted using transparent PET membrane 24-well 8.0 µm pore size cell culture inserts (BD Falcon, Franklin Lake, NJ.) coated with Type I collagen gel (KOKEN, Tokyo, Japan). Twenty-four hours after cells were seeded and duplicated on the inserts, cells that had not invaded into the lower surface of the filters were removed from the upper surface of the filter using cotton swabs. The remaining cells were fixed in acetone and methanol and stained with Trypan blue solution (Sigma-Aldrich). Invasion was quantified by visually counting photographed cells in several fields. For proliferation assays, cells were seeded in 6-well plates. After a 24-h incubation, cells were exposed to Docetaxel or DMSO as control for 24–48h. Cells were subsequently trypsinized and counted using Cell Counter model R1 (Olympus).

Immunohistochemical analyses

Because of the blinded nature of the study, pathologists at Tokyo Medical and Dental University Hospital examined 15 surgical specimens prepared from patients with squamous cell carcinoma of the tongue. Ten 400x microscopic fields containing tumor budding sites were randomly chosen in each case. If a significant decrease in EHF expression was observed in the small clusters of cancer cells at the invasive front in at least one 400x-microscopic field, the case was classified as positive for EHF downregulation. Mode of cancer invasion was assessed according to Anneroth's criteria. All studies were conducted using protocols approved by the Ethics Committee of Tokyo Medical and Dental University (D2014-084).

Human correlative studies

Publicly available datasets generated from non-cancerous and cancerous tissues from patients with HNSCC (GSE9844, GSE31056, GSE56142, and GSE37991) were used to compare EHF expression levels. Based on the *EHF* mRNA expression levels in each sample, the log2 median-centered intensity of *EHF* was calculated. The Kaplan-Meier survival curve was based on *EHF* expression of the top and bottom 30% of patients with HNSCC from the HNSCC dataset in the OncoLnc database. GraphPad Prism was used for graphs and statistics. Kaplan-Meier analysis and log-rank tests were applied for survival analysis.

Experimental calvaria invasion model

HSC3 and HSC4 cells (3×10^5 cells/mouse) were injected into the periosteal region of the parietal bone in six athymic mice as previously described [6]. Four weeks after transplantation, the samples were dissected, and

fixed with 10% neutral buffered formalin (Wako Pure Chemical Industries Ltd, Osaka, Japan), followed by H&E staining after decalcification with 10% formic acid. These experiments were performed in a blind ed manner at the Department of Pathology of Tokyo Medical and Dental University under review and approval by the Animal Care and Use Committee.

Experimental pulmonary metastasis model

A total of 2×10^6 143B-Luc cells/mouse were suspended in PBS and injected intravenously into the lateral tail vein of 8-week-old male BALB/c-nu/nu (nude) mice. After 2 weeks, mice were anesthetized with isoflurane. D-luciferin (Promega) potassium salt was injected intravenously (200 mg/kg in PBS), followed by measuring the emission intensity using the IVIS Lumina imaging system (SPI Co., Ltd. Japan). These experiments were performed in a blinded manner at the Department of Orthopaedic Surgery in the University of Yamanashi. The experimental procedures were reviewed and approved by the Animal Care and Use Committee of the Faculty of Medicine at the University of Yamanashi.

Intracardiac experimental metastasis model

MDA-MB-231-Luc cells (1×10^5) were suspended in 200 µl sterile DMEM containing 10% FBS and injected into the left ventricle of mouse hearts with a 26-gauge needle. Five weeks later, the mice were anesthetized and D-luciferin potassium salt was injected intravenously (200 mg/kg in PBS). Ten to fifteen minutes after luciferin injection, the intensity of the bioluminescence signal was measured for 1–60 s once a week using the Night OWL II LB983 system (Berthold Technologies). Imaging analyses were performed with the IndiGO2 software (Berthold Technologies). All values are shown as photons per second. These experiments were performed in a blinded manner at the Department of Molecular Pathology at the University of Tokyo. All experiments were approved by and carried out according to the guidelines of the Animal Care and the Use Committee of the Graduate School of Medicine, The University of Tokyo.

Genomic DNA sequencing

Samples from 15 patients diagnosed with an tongue cancer were obtained from Saitama Medical University International Medical Center. Written informed consent was obtained from all patients and the study protocol was reviewed and approved by the internal review board of Saitama Medical University. Genomic DNA was extracted from tongue cancer patient specimens by microdissection and with a Maxwell RSC DNA FFPE Kit (Promega, Madison, WI.) followed by next generation sequencing using Ion Torrent PGM (ThermoFisher, Waltham, MA.). These experiments were performed in a blinded manner at the Department of Pathology of Saitama Medical University International Medical Center.

Statistical analysis

The data are presented as mean ± SD. Statistical analyses were performed using Student's *t*-test between any two groups.

Supplementary References

1 Shirakihara T, Horiguchi T, Miyazawa M, Ehata S, Shibata T, Morita I *et al*. TGF-β regulates isoform switching of FGF receptors and epithelial-mesenchymal transition. *EMBO J.* 2011; 30: 783-795.

2 Horiguchi K, Sakamoto K, Koinuma D, Semba K, Inoue A, Inoue S *et al*. TGF-β drives epithelial-mesenchymal transition through δEF1-mediated downregulation of ESRP. *Oncogene* 2012; 31: 3190-3201.

3 Pfaffl MW, Tichopad A, Prgomet C, Neuvians TP. Determination of stable housekeeping genes, differentially regulated target genes and sample integrity: BestKeeper--Excel-based tool using pair-wise correlations. *Biotechnol Lett.* 2004; 26: 509-515.

4 Johnson G, Nolan T, Bustin SA. Real-time quantitative PCR, pathogen detection and MIQE. *Methods Mol. Biol.* 2013; 943: 1-16.

5 Taylor S, Wakem M, Dijkman G, Alsarraj M, Nguyen M. A practical approach to RT-qPCR-Publishing data that conform to the MIQE guidelines. *Methods* 2010; 50: S1-5.

6 Nakamura R, Kayamori K, Oue E, Sakamoto K, Harada K, Yamaguchi A. Transforming growth factor-beta synthesized by stromal cells and cancer cells participates in bone resorption induced by oral squamous cell carcinoma. *Biochem. Biophys. Res. Commun.* 2015; 458: 777-782.

Supplementary Figures

S1. *ZEB2* mRNA levels in HNSCC cells and bioinformatics analyses using the TCGA dataset. (**A**) *ZEB2* mRNA levels in HNSCC cells were determined by RT-qPCR analyses. The ratio of each mRNA to *GAPDH* in SAS cells is indicated as "1." (**B**) In cancerous tissues from patients with HNSCC in the TCGA dataset, *EHF* mRNA levels were compared with *ETS1* mRNA levels. (**C**) *EHF* mRNA levels in cancerous tissues from patients with HNSCC in the TCGA dataset were compared with those in non-cancerous tissues from a healthy donor. (**D**) Kaplan-Meier survival curve for 298 patients with HNSCC with high (red) or low (blue) EHF expression in tumor tissue in publicly available datasets are shown. EHF expression data were exported from the OncoLnc database. We used a log-rank test to analyze the survival data. p values were determined by Student's t-test. *$p < 0.01$.

S2. Evaluation of EHF-SF function in HNSCC cells. (**A**) Upon transfection with control siRNA (cont.) and EHF siRNAs (siEHFs), endogenous EHF protein levels were determined using HOC313 cell lysates. OTC04 cells were used to compare endogenous levels of EHF-SF with those in HOC313 cells. (**B**) ELF3 expression levels in HNSCC cells were determined by IB. (**C**) OBC01 cells transfected with *EHF*-specific siRNAs (siEHFs) were subjected to IB with the indicated antibodies. (**D, E, F, and G**) COS7 cells were transfected with the indicated plasmids, followed by IB (D, F, and G) and immunofluorescence analysis using an anti–Flag antibody (E). Leptomycin B (LMB) was incubated for 16 h before immunofluorescence analysis (E) or cell extraction (G). α-tubulin was used as a loading control (A, B, and C).

S3. Effect of MG132 on EHF protein stability. (**A**) TSU cells infected with control (cont.) or EHF-LF were treated with 20 μM MG132 for 10 h, followed by IB (top panel) and RT-qRCR (bottom). (**B**) OBC01 cells were treated with 20 μM MG132 for 10 h, followed by IB analyses. (**C**) TSU cells infected with either control (cont.) or EHF-SF were treated with 20 μM MG132 for 10 h, followed by IB (top panel) and RT-qRCR (bottom). (**D, E, and F**) HOC313 and TSU cells infected with lentiviruses carrying control or EHF-SF were subjected to motility assays using a Boyden chamber assay (D) and a wound healing assay (E), and chemo-resistance assays (F) in the presence of the indicated concentration (conc.) of docetaxel. (**G**) HSC3 cells were infected with lentiviral vectors encoding either control or EHF-SF, and subjected to in vitro cell proliferation analyses. (**H**) MDA-MB-231 cells infected with lentiviruses carrying control or EHF-SF were subjected to chemo-resistance assay in response to the indicated concentration (conc.) of docetaxel. Each value represents the mean ± s.d. of triplicate determinations from a representative experiment. Similar results were obtained in at least three independent experiments. α-tubulin was used as a loading control (A, B, and C).

S4. Effect of EHF mutants on ETS1 degradation. (**A**) HSC2 cells were transfected with either control siRNA (NC) or *EHF*-specific siRNAs (#2 and #5), followed by IB. **(B)** COS7 cells were transfected with control vector (cont.) or the indicated plasmids, followed by IB (top panel) and luciferase assay (bottom panel). Luciferase activity of cells transfected with the control vector is indicated as "1." α-tubulin was used as a loading control (A and B).

S5. Genomic mutation in human patients with oral cancer. Schematic illustration of the EHF protein is shown (top panel). The ETS domain (ESE domain, see Discussion) of EHF is encoded by exon 8 and exon 9. Sequencing was performed in the region shown by the red dotted line. Differentiation of oral cancer was diagnosed by pathologists. N.D., not detected.

S6. Role of EHF in degradation of ETS proteins (**A, and B**) Forty-eight h after transfection with the indicated plasmids, IB analyses were performed. The cells were treated with 20 μM MG132, 10 μM Lactacystin (Lact.), 10 μM MEK1/2 inhibitor (U0126), 10 μM CaMK-II inhibitor (KN93), 10 μM M Src inhibitor (PP1), 1 μM PKC inhibitor, Gö 6983 (GO), and 25 μM chloroquine for 10 h (B). The ratio of ETS1 to GFP in the absence of ihhibitors is indicated as "1"(bottom) (B). (**C**) OBC01 cells transfected with control siRNA (NC) or EHF siRNAs (siEHFs, #2 and #5) were treated with 20 μM MG132 for 10 h and subjected to IB with the indicated antibodies. The ratio of ETS1 to α-tubulin in control cells is indicated as "1"(bottom). α-tubulin was used as a loading control.

Supplementary figure S1

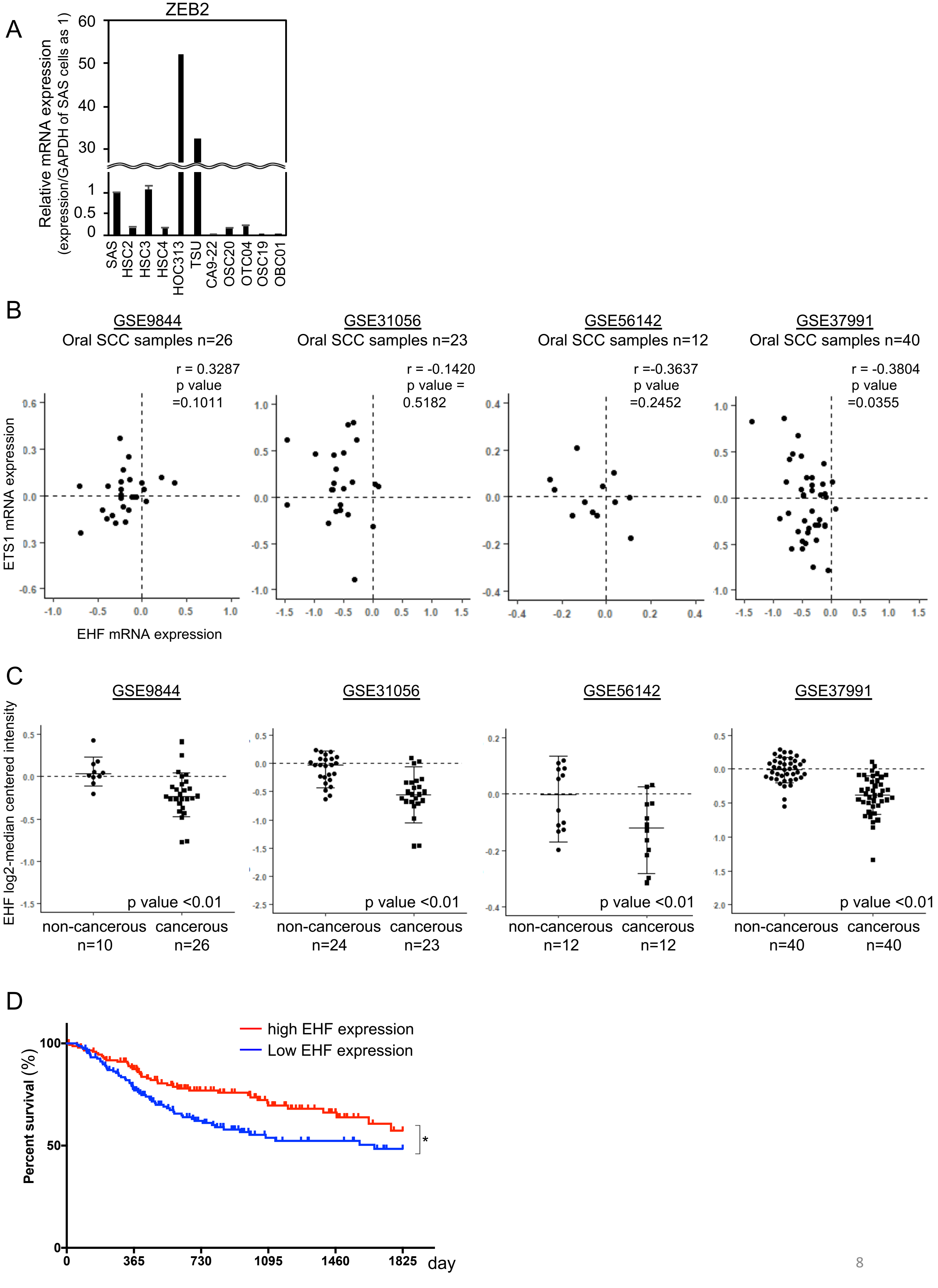

Supplementary figure S2

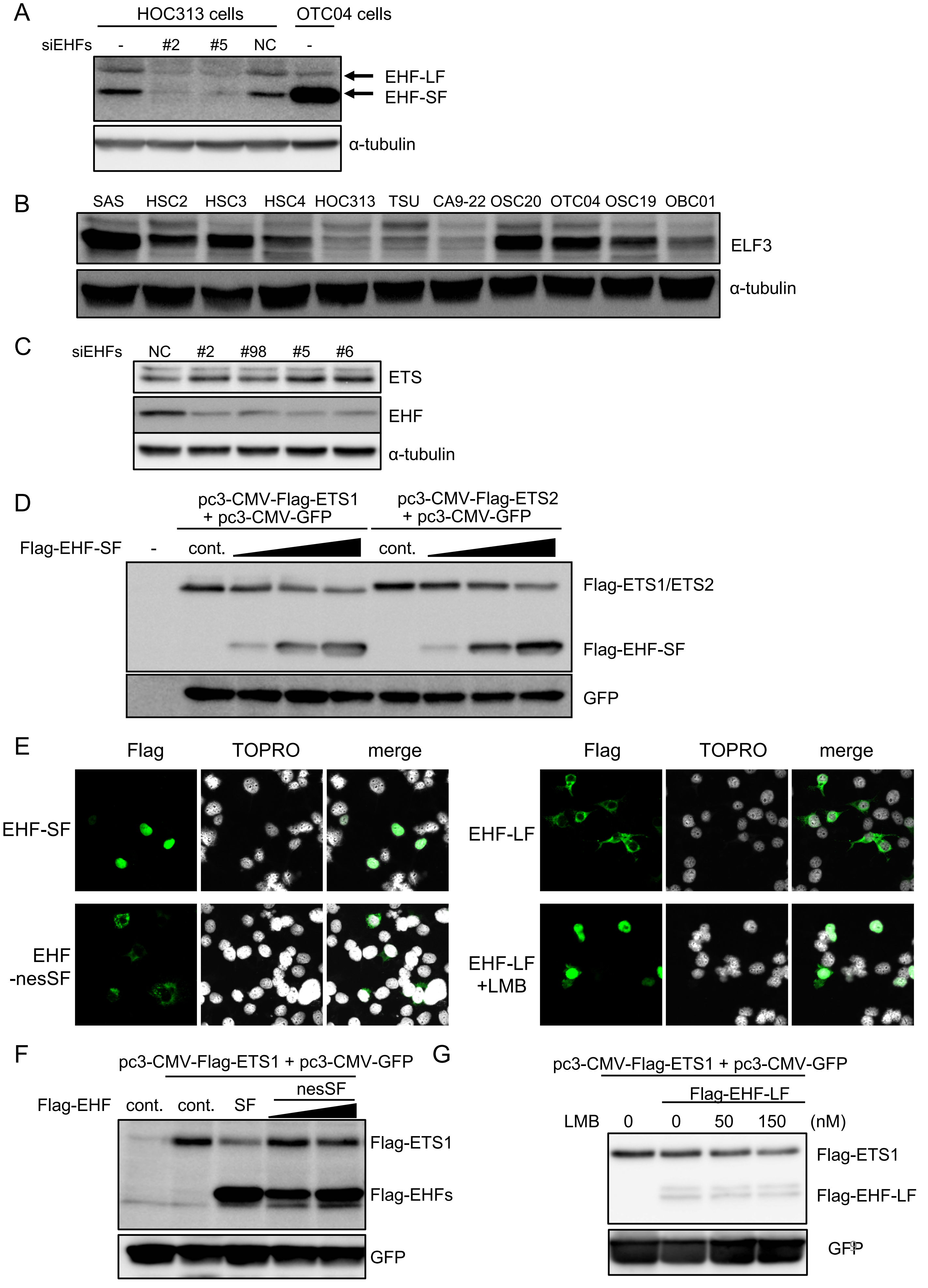

Supplementary figure S3

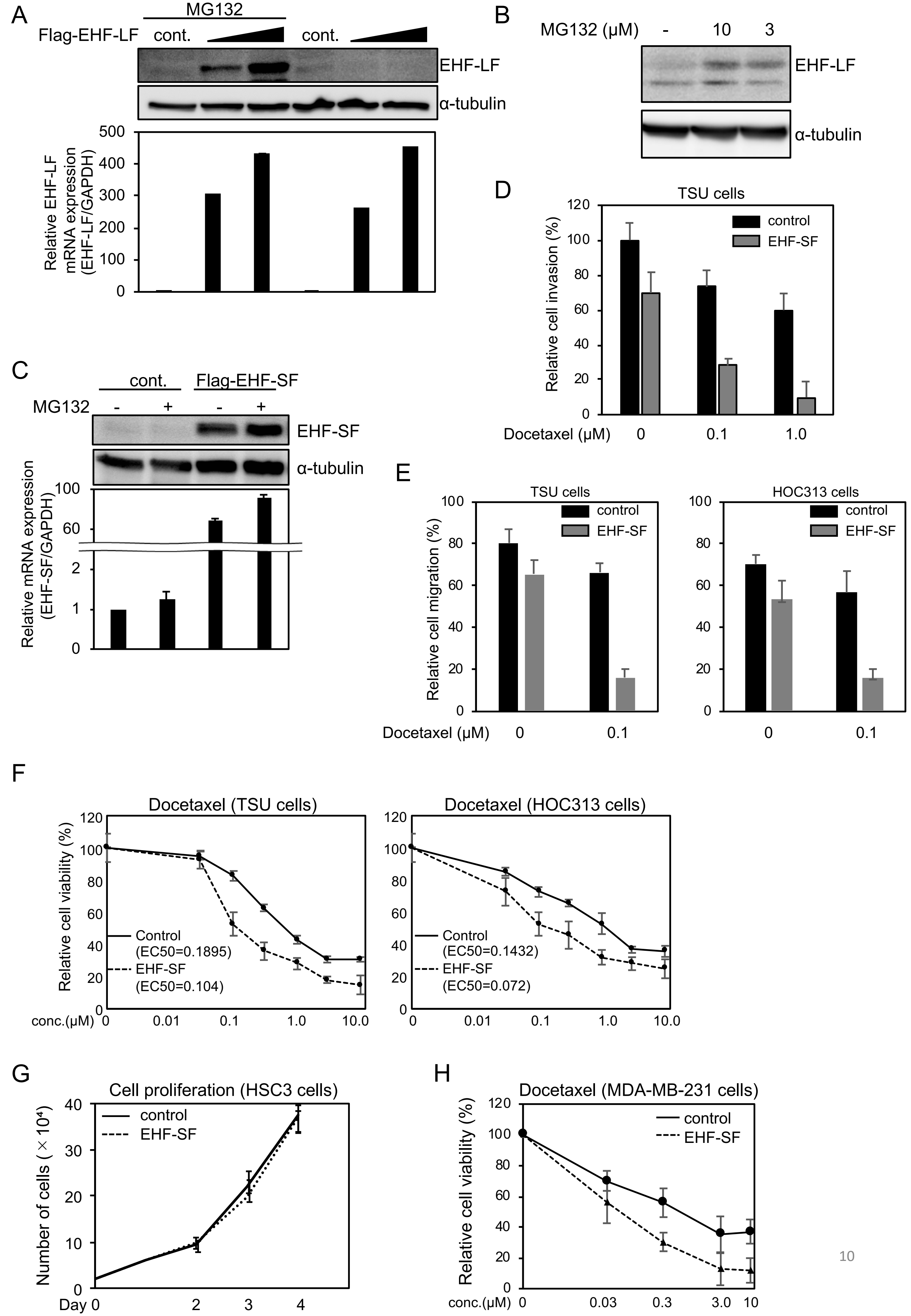

Supplementary figure S4

A

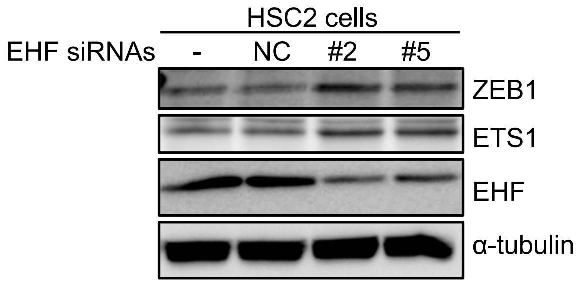

B

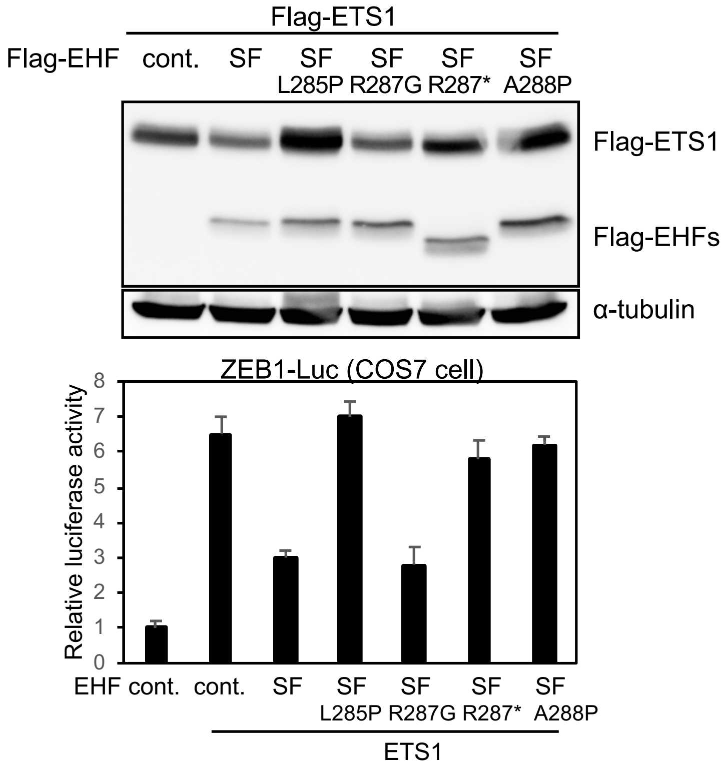

Supplementary figure S5

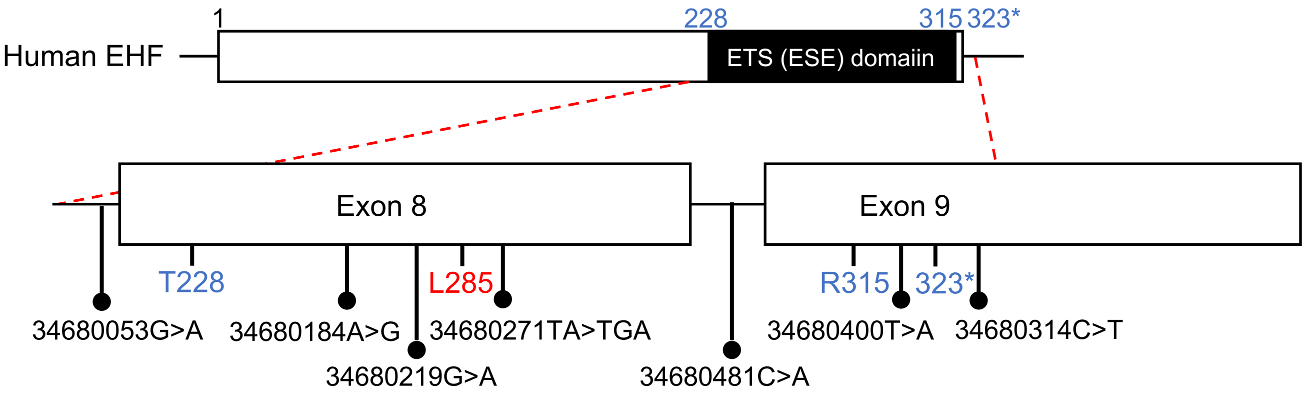

Oral SCC	region	Differentiation	*EHF* gene mutation							*ELF3* gene mutation
TK-TG15	oral tongue	moderate	34680053	G	A	Heterozygous	intron			N.D.
			34680400	T	A	Heterozygous	L298	Silent		
TK-TG14	oral tongue	moderate	N.D.							N.D.
TK-TG13	oral tongue	well	34680400	T	A	Heterozygous	L298	Silent		N.D.
TK-TG12	oral tongue	moderate	N.D.							N.D.
TK-TG11	oral tongue	spindle/poor	N.D.							N.D.
TK-TG10	oral tongue	poor	34680400	T	A	Heterozygous	L298	Silent		N.D.
TK-TG9	oral tongue	spindle cell	34680053	G	A	Heterozygous	intron			N.D.
			34680400	T	A	Heterozygous	L298	Silent		
TK-TG8	oral tongue	well	N.D.							N.D.
TK-TG7	oral tongue	poor	N.D.							N.D.
TK-TG6	oral tongue	well	N.D.							N.D.
TK-TG5	oral tongue	well	34680481	C	A	Heterozygous	intron			N.D.
TK-TG4	oral tongue	spindle cell	34680400	T	A	Heterozygous	L298	Silent		N.D.
TK-TG3	oral tongue	spindle cell/sarcomatoid	N.D.							N.D.
TK-TG2	oral tongue	poor	34680400	T	A	Heterozygous	L298	Silent		N.D.
TK-TG1	oral tongue	poor	34680184	A	G	Heterozygous	R260G	Missense		N.D.
			34680219	G	A	Heterozygous	W271*	Nonsense		
			34680271	-	G	Heterozygous	A288	Insertion		
			34680314	C	T	Heterozygous	3'non-coding			

Supplementary figure S6

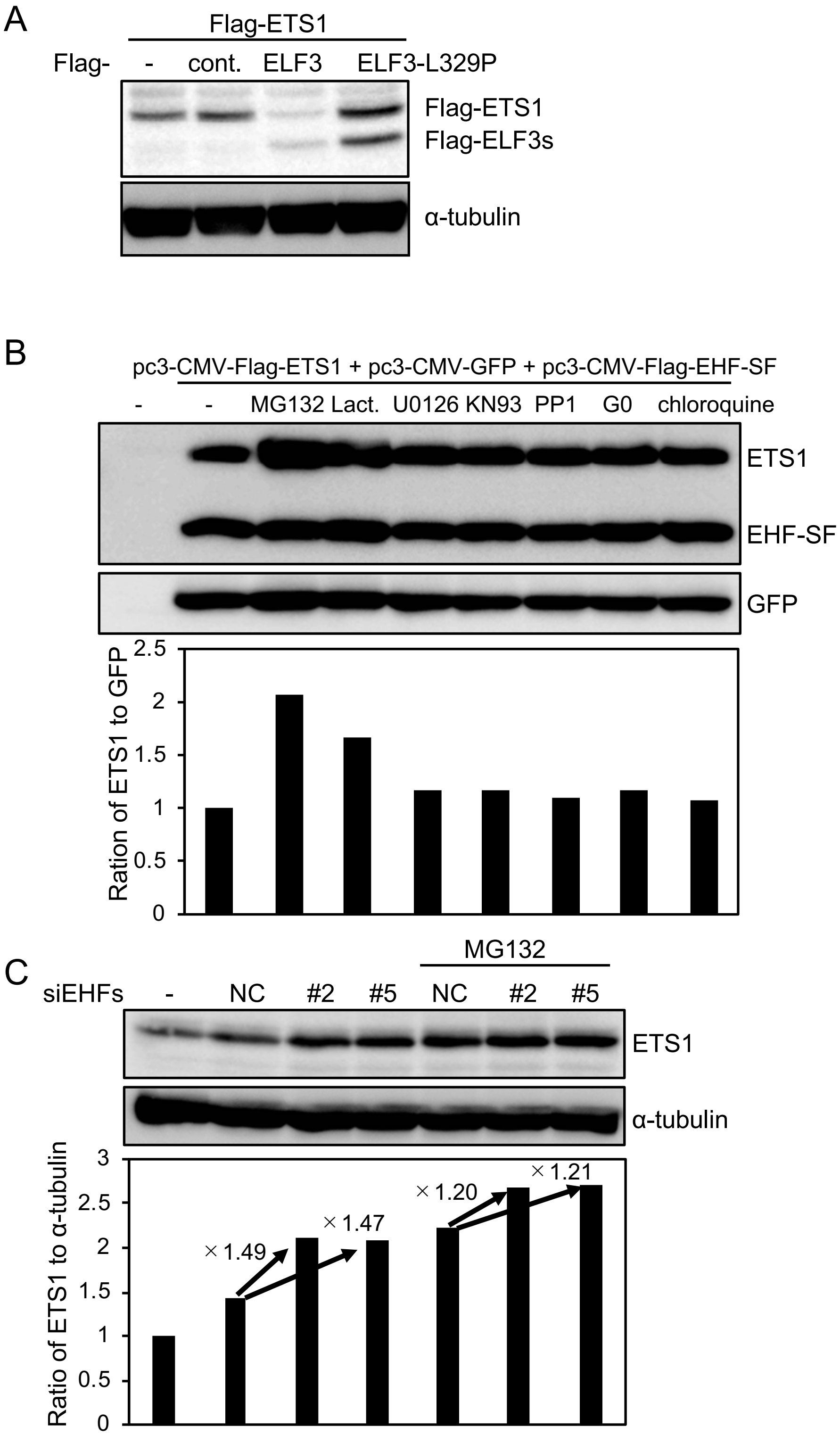

Table 1

conventional PCR perimer

	forward	reverse
hTotal-EHF-RT	5'-tgacctgttccagtccacac-3'	5'-gggttcttgtctgggttcaa-3'
hSF-EHF-RT	5'-tattggatttcccacccaga-3'	5'-ccactcccacacctggtact-3'
hLF-EHF-RT	5'-ccggagagaagaggattggt-3'	5'-ttgcacgtggagtagctgtc-3'

quantitative RT PCR perimer

	forward	reverse
hZeb1-RT	5'-caatgatcagcctcaatctgca-3'	5'-ccattggtggttgatccca-3'
hETS1-RT	5'-cccgtacgtcccccactcct-3'	5'-tgggacatctgcacattcca-3'
hELF3-RT	5'-caactatggggccaaaagaa-3'	5'-tccaggatctcccgtttgta-3'
hELF5-RT	5'-gttctgctgcgaccagtaca-3'	5'-tgccacttgttttcaagcag-3'
hEHF-RT	5'-tgacctgttccagtccacac-3'	5'-gggttcttgtctgggttcaa-3'
hEHF-LF-RT	5'-ccggagagaagaggattggt-3'	5'-ccactcccacacctggtact-3'
hGAPDH-RT	5'-cgaccactttgtcaagctca-3'	5'-cccgtacgtcccccactcct-3'

cloning primer

	forward	reverse
hEHF LF	5'-ttgaattcatggggttgccggag-3'	5'-ttctcgagtcagttttcattttc-3'
hEHF SF	5'-ttgaattcattctggaaggaggt-3'	5'-ttctcgagtcagttttcattttc-3'
hELF5	5'-tctgagaagggttcagaagc-3'	5'-ccataaaatgagcttgatgc-3'
hEHF SF pointed	5'-ttgaattcatggggttgccggag-3'	5'-aacccgagagggtgactcgagtt-3'
hEHF ETS domain	5'-ttgaattcactcacttatgggag-3'	5'-gaaaatgaaaactgactcgagtt-3'

mutagenesis primer

	forward	reverse
hESE3 E233E	5'-actcacttatgggagttcatccgcgacat-3'	5'-atgtcgcggatgaactcccataagtgagt-3'
hESE3 P285L	5'-tgacctatgaaaagctcagccgagctatgag-3'	5'-ctcatagctcggctgagcttttcataggtca-3'
hESE3 R287G	5'-tatgaaaagctcagcggagctatgagatatt-3'	5'-aatatctcatagctccgctgagcttttcata-3'
hESE3 A288P	5'-gaaaagctcagccgacctatgagatattact-3'	5'-agtaatatctcataggtcggctgagcttttc-3'
hESE3 M23V	5'-attcttttcaagatcgtgattctggaaggag-3'	5'-ctccttccagaatcacgatcttgaaaagaat-3'
hEHF-R287stop	5'-tatgaaaagctcagctgagctatgagatatt-3'	5'-aatatctcatagctcagctgagcttttcata-3'
hEHF-D208H	5'-gttgcagagtcacctcatatgaaaaaggagc-3'	5'-gctcctttttcatatgaggtgactctgcaac-3'
hESE3 K21R	5'-ctgaaattcttttcaggatcatgattctgga-3'	5'-tccagaatcatgatcctgaaaagaatttcag-3'
hESE3 R6G-L9V	5'-ccggagggaagaggagtggtcctgcttttaa-3'	3'-ttaaaagcaggaccactcctcttccctccgg-3'
